# Advancing Social Life Cycle Assessment: A Novel Approach to Uncertainty Analysis

**DOI:** 10.1021/acs.iecr.5c03058

**Published:** 2026-01-26

**Authors:** Beatriz Cassuriaga, Andreia Santos, Ana Carvalho

**Affiliations:** CEGIST − Centro de Estudos de Gestão do Instituto Superior Técnico, 37809 Universidade de Lisboa, Av. Rovisco Pais, Lisboa 1049-101, Portugal

## Abstract

Social life cycle assessment is gaining importance, being recognized as a well-established methodology to evaluate potential social risks that might occur in value chains. Several studies have been conducted in applying traditional social databases (e.g., Social Hotspot Database) to assess social risks, but these studies generally do not consider the uncertainty associated with the characterization factors used in the models. This type of uncertainty is intrinsic to social risk modeling, as the underlying indicators and expert-based assessments are inherently variable. Therefore, this paper aims to address this literature gap by proposing an uncertainty analysis methodology that explicitly accounts for the uncertainty associated with the characterization factors. It represents one of the first studies to model such uncertainty directly within the context of the Social Life Cycle Assessment. The methodology will be applied to assess the social performance of two components, a car dashboard and a ship counter bar, manufactured using conventional materials (ABS and reinforced gypsum) and an innovative cellulose-based material. The results show that the methodology is easily employed and applicable to different case studies. The cellulose-based material exhibited significantly lower potential social impacts in the ship counter bar and consistently higher impacts in the car dashboard when compared to conventional materials, and these findings remained consistent even when accounting for uncertainty in the characterization factors. The approach also quantifies the confidence associated with each comparison, reinforcing the robustness of the conclusions. By integrating uncertainty modeling into the Social Life Cycle Assessment, the study enhances the transparency and interpretability of social performance evaluations across different value chains.

## Graphical abstract

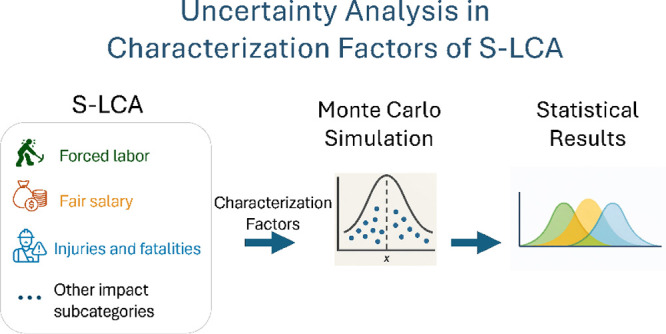

## 1 Introduction

Social Life Cycle Assessment (S-LCA) has become a relevant approach to identify and compare social impacts across the life cycle of products and materials. Over the past decades, S-LCA has undergone significant methodological development, with a steady increase in the number of published studies, formalization of guidelines, and improvement of assessment frameworks and indicators.^1,2^

S-LCA is a valuable tool for decision-making, for instance, when comparing two product systems, guiding responsible sourcing and supplier selection, enhancing management systems, supporting responsible investment choices, improving product design, or optimizing existing processes.^3^

The Social Hotspots Database (SHDB) is among the most widely used databases for assessing S-LCA.^2^ It provides a global framework for identifying potential social risks along supply chains based on country- and sector-level data, allowing practitioners to focus data collection efforts where risks are most significant.^4^ However, several authors have pointed out limitations related to data transparency, temporal coverage, and representativeness, since many SHDB indicators rely on aggregated national statistics that may not fully capture sector- or company-specific conditions.^2,5^ Despite these constraints, the SHDB remains one of the most established and consistently applied databases in S-LCA research, supporting methodological alignment and comparability with previous studies.^2,4,6^ Alternative databases, such as PSILCA, offer broader coverage and more recent data sources,^7^ but their higher level of granularity and modeling complexity introduce additional structural uncertainty that is not required for the present study. Since the aim here is methodological development focused on the characterization and propagation of uncertainty in characterization factors, a more aggregated and stable structure, such as that of the SHDB, was more appropriate for isolating the effects of the proposed method. To address the limitations associated with data quality and representativeness, a systematic Data Source Quality Assessment based on the Product Environmental Footprint (PEF) criteria was conducted for all SHDB indicators, ensuring a transparent and structured evaluation of uncertainty.

These uncertainties are amplified by the qualitative and heterogeneous nature of social information, which often relies on expert judgment, secondary data, and diverse methodological assumptions. Consequently, most S-LCA studies are still conducted deterministically, overlooking the uncertainty associated with social data and characterization factors,^8^ which compromises the interpretability and reliability of the results.^5^

In this context, the explicit treatment of uncertainty has been identified as a major research gap in S-LCA.^9,10^ Although uncertainty analysis is increasingly recognized as an essential component of Environmental LCA, its practical application remains limited, with less than 20% of environmental LCA studies published since 2014 reporting uncertainty analysis.^11^ Within S-LCA, this issue becomes even more critical, as very few studies have examined uncertainty in the social dimension. Early efforts to incorporate stochastic approaches include,^12^ who applied Monte Carlo simulation to evaluate epistemic uncertainty in the construction of a Social Vulnerability Index, showing how methodological choices such as indicator selection and weighting influence the reliability of social results. Do Carmo et al. (2017)^8^ were among the first to apply Monte Carlo simulation directly in S-LCA, demonstrating its potential to address uncertainty related to subjective judgments, weights, and social indicators, while Carreira-Barral et al. (2025)^13^ extended this probabilistic approach to inventory-level uncertainty using SimaPro software.

More recently, hybrid- and fuzzy-based approaches have been introduced to capture the qualitative and ambiguous nature of social information. Fidan et al. (2021)^14^ combined the Subcategory Assessment Method (SAM) with a hesitant fuzzy AHP model to account for stakeholder hesitation in multidimensional sustainability assessment; Moktadir and Ren (2025)^15^ proposed a trapezoidal fuzzy LBWA–MABAC framework to integrate subjective expert judgments with PSILCA-based data in S-LCA; Tokede (2025)^16^ applied the Intuitionistic Fuzzy Set (IFS) theory to manage linguistic ambiguity and missing information in Social Life Cycle Impact Assessment; and Villalba et al. (2025)^17^ developed a hybrid fuzzy DEMATEL–DANP–TOPSIS model to evaluate trade-offs among social, environmental, and economic criteria under uncertainty.

Together, these studies illustrate that the literature has begun to acknowledge uncertainty in social assessment, using either fuzzy or stochastic methods. However, most approaches focus on qualitative representation or inventory-level uncertainty, and none explicitly model the uncertainty embedded in social characterization factors or database parameters that determine impact calculation. This focus is particularly relevant because social characterization factors play a central role in S-LCA: they convert social inventory data into quantitative impact results and therefore largely determine the magnitude and direction of social performance outcomes. These factors are derived from secondary databases such as the SHDB or PSILCA, which rely on aggregated socioeconomic statistics and fixed multipliers that implicitly define relative weightings among risk levels. Such assumptions, while practical, are not empirically validated and introduce structural uncertainty into the impact assessment. Ignoring the uncertainty associated with these parameters can lead to a false sense of precision and compromises the reliability of comparative assessments. By explicitly modeling uncertainty in social characterization factors, this study provides probabilistic results that reflect the uncertainty and robustness of the assessment. Moreover, this approach aligns S-LCA practice with well-established standards in environmental LCA, contributing to more transparent and statistically sound decision support in sustainability assessments.

Given this context, this study aims to propose an uncertainty analysis methodology that explicitly incorporates uncertainty in S-LCA model (e.g., defined parameters such as CFs) through a stochastic approach based on Monte Carlo simulations. To this end, a methodology originally developed by Santos et al. (2022)^18^ in the field of Environmental Life Cycle Assessment (LCA) is adapted and extended to the context of S-LCA, focusing on the propagation of uncertainty in CFs. The literature recognizes Monte Carlo simulation as a robust tool for quantifying uncertainty in LCA studies, contributing to more reliable and decision-relevant outcomes.^19,20^

It is important to emphasize that the purpose of uncertainty modeling in this context is not to correct or eliminate data gaps but to make them explicit and interpretable. As highlighted by Riedmaier et al. (2020),^21^ uncertainty quantification enhances model credibility by clarifying the confidence and uncertainty underlying results, while Soize (2017)^22^ argues that probabilistic modeling remains appropriate under epistemic uncertainty when the objective is to represent plausible variability and inform decision-making rather than achieve statistical precision. This perspective underlies the approach proposed here, reinforcing the role of uncertainty modeling to improve transparency and the interpretability of S-LCA outcomes.

The new methodology proposed in this paper is applied to two comparative cases, with the specific objective of evaluating and contrasting the social performance of components (a car dashboard and a ship counter bar) manufactured using conventional materials (ABS and reinforced gypsum) and an innovative cellulose-based material developed. The analysis does not focus on identifying the most critical subcategories after applying uncertainty; instead, it emphasizes a comparison between material alternatives under controlled uncertainty conditions.

The main scientific contribution of this study lies in the new methodology, based on a stochastic approach for S-LCA, which integrates uncertainty analysis into the social CFs, being among the first studies to explicitly integrate the inherent uncertainty of social databases and characterization models into S-LCA. Additionally, the methodology is implemented in Excel and @Risk, representing a key advantage for its adoption in professional and institutional contexts outside academia. The literature emphasizes that the availability of simple, familiar, and low-cost tools supports the practical integration of sustainability assessment methods in companies and technical organizations, broadening their reach and utility.^18,23^

This Account is structured as follows: the next section presents the adapted methodology, detailing the adjustments required to integrate uncertainty into social data. Then, the results of the deterministic and stochastic analyses are discussed, enabling a comparison and validation of the consistency of the conclusions under different uncertainty scenarios. Finally, the practical and scientific implications of the findings are explored, highlighting the potential of this approach to support more responsible decisions in the design and selection of sustainable materials.

## 2 Methodology

This study proposes a new methodology, which extends and adapts the methodology developed by Santos et al. (2022),^18^ originally designed to address the uncertainty of CFs in Environmental Life Cycle Assessment (LCA). The original methodology cannot be directly applied to social life cycle assessment due to the distinct nature of social indicators and CFs. Therefore, this study involved a substantial rethinking and adjustment of the original approach to ensure its applicability to S-LCA. The adapted methodology consists of six main steps (Figure 1): Define the Goal and Scope (Step 1); Model the Systems (Step 2); Select the Impact Assessment Method (Step 3); Conduct a Deterministic Impact Assessment (Step 4); Conduct a Stochastic Impact Assessment (Step 5); and Analyze and Compare the Deterministic and Stochastic Results (Step 6).

**Figure 1:**
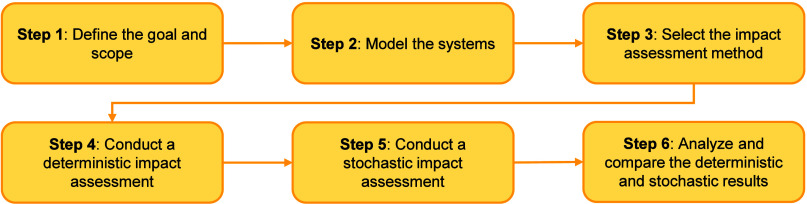
Methodology for integrating the uncertainty in the CFs into social life cycle assessments

(adapted from Santos et al.[Bibr ref18] (2022)).

Below, each of these steps is explained, highlighting the adjustments required for their application to S-LCA.

### Step 1: Define the Goal and Scope

The first step involves defining the goal of the life cycle assessment (LCA) and characterizing the system(s) under study (i.e., scope) through the definition of the system boundary and functional unit.

The goal of the study presented in this paper is to assess and compare the social performance of two components (a car dashboard and a ship counter bar) made with two material alternatives. In the case of the car dashboard, a version of this component made using a conventional material, specifically, acrylonitrile butadiene styrene (ABS) plastic, is compared with a version made using a cellulose-based material.^24^ In the case of the ship counter bar, a version of this component made by using gypsum reinforced with fiberglass (a more conventional material) is compared with a version made by using the same cellulose-based material. Although the material is a composite of gypsum and fiberglass, it is referred to as ‘gypsum’ throughout this study for simplicity. The comparisons presented in this study will support material selection in design and production decisions in the automotive and maritime sectors by identifying social risks along the life cycle of each alternative.

The system boundary defines which stages of the system’s life cycle will be considered.^25^ In this study, the system boundary is defined as cradle-to-grave, covering the stages from raw material extraction to final disposal, including intermediate processing, manufacturing, distribution, and use.^24^ Figure 2 illustrates the main life cycle stages considered for the systems under comparison, highlighting the flow of materials and key processes included within the cradle-to-grave boundary.

**Figure 2:**
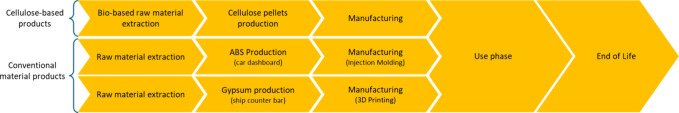
Life cycle of cellulose-based material products and conventional alternative products (ABS and gypsum).

The functional unit serves as a quantitative basis for all social impacts calculated during the impact assessment (Step 4). The functional unit used in this study to ensure comparability between systems is one square meter of the final component, with equivalent thickness and a service life of 10 years for all alternatives. The service life of 10 years was adopted as a reference period based on the average lifetime of the automotive application and on the typical maintenance and refurbishment intervals observed in maritime applications, rather than on the intrinsic durability of the materials themselves.^26–28^ In both contexts, components do not remain in use beyond the functional lifetime of the systems in which they operate, and the 10 year period reflects this effective functional interval. The cellulose-based components are assumed to remain functional for at least this same period, providing a consistent basis for measuring and comparing the social impacts of the materials under analysis.

### Step 2: Model the Systems

In the second step, the systems under study are modeled. This modeling can be accomplished using an LCA software and allows quantifying the social flows (e.g., worker hours with high risk of child labor) associated with the systems. To quantify these social flows, data on different social issues are required, which can be acquired using different sources such as databases. In this study, the four systems under study (i.e., ABS car dashboard, cellulose-based car dashboard, gypsum ship counter bar, and cellulose-based ship counter bar) were modeled in SimaPro^29^ using the SHDB.^30^ This database provides information on social risks across 244 regions and 57 sectors, using a wide range of social impact indicators (e.g., unemployment percentage in each country and sector) derived from multiple sources, including country statistics, academic research, nongovernmental organizations’ reports, and intergovernmental databases.^31^ To model a system using SHDB, the following steps are required:

- Identify materials, utilities, and processes: All the materials, utilities, and processes involved within the system boundary defined in Step 1 need to be identified, together with the respective quantities of materials and utilities (e.g., mass, energy);
- Assign each material, utility, and process to the corresponding economic sector and region: The economic sector to which these materials, utilities, and processes belong can be identified using the global economic equilibrium model indicates 57 economic sectors across 140 regions,^32^ which are then available in the SHDB. Then for each material, it is required to identify the region (e.g., country) from where they are sourced or occur needs to be defined. By using this information, the model can be developed in SimaPro with the support of the SHDB. The model will identify all economic sectors and regions linked to the sector/region defined for each material, utility, and process.
- Collect cost data for each material, utility, and process: Another information required to model a system using the SHDB in SimaPro is the cost associated with the different materials, utilities, and processes involved within the system boundary defined in Step 1. Using this information, it is possible to determine the total worker hours required by each material, utility, and process since the SHDB has information on worker hours per US dollar for all 57 economic sectors in the 140 regions stated.

The data collected for the ABS car dashboard system are presented in Tables 1 and 2. Due to confidentiality restrictions, the information related to quantities and economic values is expressed in percentage form rather than absolute figures.

**Table 1:** Primary Data Collected for the ABS Car Dashboard Manufacturing Stage

material	sector	% qty	% cost	country
**ABS Car Dashboard**				
acrylonitrile butadiene styrene (ABS) copolymer	chemical products	52.1%	93.83%	Italy
water	water	0.34%	0.36%	Italy
kaolin	manufacture nonmetallic mineral products	0.1%	0.01%	Italy
lime	manufacture nonmetallic mineral products	0.12%	0.01%	Italy
lubricating oil	chemical products	0.09%	0.44%	Italy
malusil	chemical products	0.01%	0.03%	Italy
polyethylene	chemical products	0.05%	0.04%	Italy
polypropylene	chemical products	0.11%	0.11%	Italy
solvent, organic	chemical products	1.38%	1.8%	Italy
titanium dioxide	manufacture nonmetallic mineral products	0.06%	0.04%	Italy
electricity	electricity	45.63%	3.34%	Italy

**Table 2:** Primary Data Collected for the ABS Car Dashboard End-of-Life Stage

material	sector	% qty	% cost	country
**ABS Car Dashboard**				
landfill	water	24.9%	19.92%	Italy
incineration	water	32.5%	18.21%	Italy
recycling	water	42.6%	61.88%	Italy

In the SHDB, end-of-life processes such as landfill, incineration, and recycling are represented under the “Water” sector, as defined by the GTAP framework.^32^ This sector includes water supply, sewerage, waste management, and remediation activities and is therefore commonly used as the reference for modeling end-of-life treatments to ensure consistency with the SHDB and GTAP classifications.

The data collected for the other three systems under study are presented in the Supporting Information (Tables S1 to S6). All data are aligned with the functional unit defined in Step 1, ensuring consistency and comparability across the four systems. The output of this step is an inventory list of social flows and the corresponding quantities that quantitatively relate to the functional unit established in Step 1.

### Step 3: Select the Impact Assessment Method

The third step of the methodology consists of selecting the impact assessment method used to convert the inventory collected in Step 2 into potential social impacts. In this study, the social flows collected in the previous step were converted into potential social impacts by using the Social Hotspot Index (SHI) method. This method evaluates 25 impact subcategories based on 39 social indicators (Table 3) and applies CFs to translate these indicators into potential social risks. These subcategories can be further grouped into five main impact categories: Labor Rights and Decent Work, Human Rights, Health and Safety, Governance, and Community.^31^

**Table 3:** Social Hotspots Index: Categories, Subcategories, Indicators, Normalization Factors (NFs), and Characterization Factors (CFs) by the Risk Level^31^

				characterization factor^a^			
impact categories	norm factor	impact subcategories	SHI measurements	LR	MR	HR	VH
Labor Rights and Decent Work	1.8	Wage Assessment	Risk that Avg Wage is Below Country Minimum Wage	0.1	1.3	6.7	13.3
			Risk that Sector Avg Wage is Below Living Wage	0.1	1.3	6.7	13.3
			Risk that Sector Avg Wage is Below Sweatered Wage	0.1	1.3	6.7	13.3
		Poverty	Percent of population living under the relevant poverty line	0.4	4.0	20.0	40.0
		Child Labor	Risk of child labor by sector (qualitative)	0.4	4.0	20.0	40.0
		Forced Labor	Overall Forced Labor in Country	0.4	4.0	20.0	40.0
		Excessive Working Time	Percent of Population working > *X* h per week. > 60 h per week	0.4	4.0	20.0	40.0
		Freedom of Association	Overall risk of Freedom of Association	0.4	4.0	20.0	40.0
		Migrant Labor	Evidence of Risk to Migrant Workers – Qualitative	0.4	4.0	20.0	40.0
		Social Benefits	Overall risk of inadequate social benefits	0.4	4.0	20.0	40.0
		Labor Laws Conventions	Number of Labor Laws by Sector	0.4	4.0	20.0	
		Discrimination	Prevalence of discrimination in the workplace (qualitative)	0.4	4.0	20.0	40.0
		Unemployment	Unemployment percentage at sector level	0.4	4.0	20.0	40.0
Health and Safety	10.0	Occupational Toxics and Hazards	Disability-adjusted life years due to occupational-related Lung Cancer	0.1	1.3	6.7	13.3
			Overall Occupational Cancer Risk – loss of life (DALYs)	0.1	1.3	6.7	13.3
			Overall Occupational Noise Exposure Risk	0.1	1.3	6.7	13.3
		Injuries and Fatalities	Fatal injuries by sector	0.2	2.0	10.0	20.0
			Non-Fatal Work-Related Injuries by Sector	0.2	2.0	10.0	20.0
Human Rights	4.0	Indigenous Rights	Indigenous Sector Issues Identified	0.2	2.0	10.0	20.0
			Overall risk of indigenous rights being infringed	0.2	2.0	10.0	
		Gender Equity	Overall Gender Inequity in Country	0.4	4.0	20.0	40.0
		High Conflict Zones	Overall High Conflict	0.4	4.0	20.0	40.0
		Non-Communicable Diseases	Overall Non-communicable Diseases and other health risks	0.4	4.0	20.0	
		Communicable Diseases	Age-standardized MRs from communicable diseases (per 100,000 population)	0.1	0.8	4.0	8.0
			Cases of HIV (per 1000 adults 15–49 years)	0.1	0.8	4.0	8.0
			Cases of Tuberculosis (per 100,000 population)	0.1	0.8	4.0	8.0
			Dengue Fever, Incidence Rate (per 100,000 population)	0.1	0.8	4.0	8.0
			Notified cases of Malaria (per 100,000 population)	0.1	0.8	4.0	8.0
Governance	10.0	Legal System	Overall Fragility in Legal System	0.4	4.0	20.0	40.0
		Corruption	Overall Corruption	0.4	4.0	20.0	40.0
Community	4.0	Access to Drinking Water	% Total Access to an Improved Source of Drinking Water	0.4	4.0	20.0	40.0
		Access to Sanitation	% Total Access to an Improved Source of Sanitation	0.4	4.0	20.0	40.0
		Children Out of School	Percent of Children Out of Primary School, total	0.4	4.0	20.0	40.0
		Access to Hospital Beds	Number of Hospital Beds per 1000 population	0.4	4.0	20.0	
		Smallholder vs Commercial Farms	Large Holdings land % < *x* hectares	0.1	1.0	5.0	10.0
			Percentage of commercially owned farms in country	0.1	1.0	5.0	10.0
			Percentage of family-owned farms in country	0.1	1.0	5.0	10.0
			Smallholdings Land % < *x* hectares	0.1	1.0	5.0	10.0
			Overall risk of Freedom of Association	0.2	1.8	9.1	18.2

a Risk level: (LR) low risk; (MR) medium risk; (HR) high risk; (VH) very high risk.

### Step 4: Conduct a Deterministic Impact Assessment

In the fourth step of the methodology, the inventory collected in Step 2 is converted into potential social impacts using the impact assessment method selected in Step 3. The first task of an impact assessment is to calculate the characterized results of the impact categories expressed in medium risk hours equivalent (MRH eq) by multiplying the inventory collected in the previous step with the CFs of each impact category (see Table 3). As previously noted, the 39 indicators are used to assess 25 impact subcategories – some evaluated using a single indicator and others using multiple indicators (Table 3). Each indicator is linked to a reference scale with four risk levels (low, medium, high, and very high), for which the SHDB provides CFs representing the likelihood of unfavorable situations (Table 3).^31^ For example, for the Child Labor impact subcategory, the characterized result in medium risk hours equivalent is calculated by multiplying the number of worker hours with low (LR), medium (MR), high (HR), and very high risk (VH) of child labor by 0.4, 4, 20, and 40 (Table 3), respectively, and adding these four results together. The characterized results of each impact subcategory can be normalized and weighted using the normalization and weighting factors provided by the SHI method. The sum of these weighted results generates a single score (SS), representing the overall potential social impact of the systems under analysis. While the use of a single aggregated score facilitates the overall comparison between alternatives, it may also mask trade-offs among individual social subcategories. Therefore, to complement the single score and capture these potential differences, the results were also analyzed at the subcategory level to provide a more detailed understanding of specific social issues. The characterization, normalization, and weighting steps required by the SHI method were conducted in Microsoft Excel,^33^ using the social flow data exported from SimaPro 8.4.0 software.^29^ The outputs of this step are the characterized, normalized, and weighted results of the 25 impact subcategories, together with the single score of each system under analysis.

### Step 5: Conduct a Stochastic Impact Assessment

The CFs considered in the SHI method are subject to uncertainty for several reasons. First, there is no available documentation explaining how these factors were derived. These factors vary according to the risk level considered (Table 3), but no rationale is provided for the fixed multipliers applied between levels, namely, that the medium risk CF is 10 times higher than the low risk CF, the high risk CF is 5 times higher than the medium one, and the very high risk CF is 2 times higher than the high risk factor. Additionally, as described in Section 2, the SHDB classifies each sector and region across four risk levels based on heterogeneous social indicators with varying reliability.^31^ As a result, the CFs used in the SHI method carry a degree of uncertainty that can affect the outcomes of S-LCA studies and should therefore be considered in the interpretation of the results. This step of the methodology provides a framework to support practitioners in incorporating the uncertainty of CFs into social life cycle assessments and interpreting the results accordingly.

To address the uncertainty associated with the CFs in Step 4, Monte Carlo simulation was selected as the stochastic modeling approach.^18^ Since the deterministic impact assessment was conducted in Microsoft Excel (Step 4), the stochastic impact assessment was performed in this study using the @Risk software, the leading Monte Carlo simulation add-in for Excel.^34^

This tool was selected due to both accessibility and methodological advantages. Excel offers a familiar and low-cost platform for modeling,^35^ which facilitates replication and dissemination of stochastic analysis beyond academic contexts. The @Risk add-in is recognized as the leading Monte Carlo simulation tool for Excel,^18,34^ enabling the implementation of probabilistic models with transparency and control over uncertainty parameters. Moreover, not all dedicated LCA software packages include uncertainty analysis modules, and when available, Monte Carlo simulations are typically limited to the Life Cycle Inventory (LCI) phase,^36,37^ without incorporating the uncertainty of characterization factors. Since this study specifically addresses uncertainty in social characterization factors, the use of Excel combined with @Risk allowed the direct application of Monte Carlo simulation to these parameters, ensuring methodological rigor and computational flexibility consistent with the approach proposed by Santos et al. (2022).^18^

The initial step in a Monte Carlo simulation involves assigning probability distributions to uncertain parameters. As previously explained, the CFs applied vary according to the risk level considered (Table 3) and the classification of each sector and region as having low, medium, high, and very high risk in each social issue is based on indicators drawn from multiple sources with varying degrees of reliability. Since the analysis of uncertainty in the characterization factors used in S-LCA, as proposed in this study, has not been previously addressed, it was necessary to adapt concepts from other fields. For this reason, the criteria used to evaluate the uncertainty of the data sources of the 39 social indicators used by the SHI method (Table 3) were taken from the Product Environmental Footprint (PEF) guidelines,^38^ the method recommended by the European Union for environmental LCA. These six criteria are completeness, methodological appropriateness, technological representativeness, geographical representativeness, temporal representativeness, and precision.^38^ Five of these six criteria (completeness, methodological appropriateness, technological representativeness, geographical representativeness, and temporal representativeness) are also suggested in the Guidelines for S-LCA for assessing data quality and, therefore, the level of uncertainty associated with the data.^1^ However, precision was also included in this study, as it corresponds to ‘parameter uncertainty’ in the ISO 14044 standard,^39^ and this research specifically addresses uncertainty in a key parameter of S-LCA (i.e., CFs).

Each data source of the 39 social indicators was scored from 1 (excellent) to 5 (very poor) in each criterion based on information provided by the SHDB documentation and the metadata associated with each source. The evaluation was conducted directly by the authors, who accessed the links to the original data sources listed in the SHDB^31^ and assessed them according to the six data quality criteria defined in the PEF method^38^ and adapted to the S-LCA context. The adapted criteria, their descriptions, and the evaluation guidance used to assign scores from 1 to 5 are presented in the Supporting Information (Appendix S2, Table S7). The scoring was performed by a single evaluator to ensure internal consistency and validated by two evaluators afterward, acknowledging that this manual assessment introduces a limited degree of subjectivity.

The full list of evaluated sources, the criteria scores, and the justifications of the classifications are presented in the Supporting Information (Table S8). An average of the six scores was calculated to define the uncertainty score of each indicator (see Supporting Information, Table S9). When an indicator was quantified using more than one source, the uncertainties of all sources used were individually assessed. The final uncertainty score of each indicator was calculated as the average of the scores across all criteria and sources. For example, the indicator “Risk of child labor by sector” used in the impact subcategory Child Labor is quantified using two data sources, the United States Department of Labor and the International Trade Union Confederation (see Supporting Information, Table S8). Each of these two data sources was classified in the six criteria, and the final uncertainty score of the “Risk of child labor by sector” indicator was calculated as the average of the 12 scores obtained from the assessment of the two data sources in the six criteria. Using the arithmetic mean assigns equal weight to all six criteria, which is a simplification adopted in this study, since the methodological scope focuses on the propagation of uncertainty in the characterization factors themselves rather than on constructing or testing a weighting model for data quality assessment. In practice, some criteria such as temporal representativeness may influence the uncertainty of the indicators more strongly than others, and this limitation can be explored in future work.

This process allowed the classification of indicators into three uncertainty levels: low (uncertainty score ≤ 1.5), medium (1.5< uncertainty score ≤ 3.0), and high (uncertainty score >3.0) (see Supporting Information, Table S8), in accordance with the uncertainty thresholds adopted in the PEF methodology.^38^ For example, the indicator “Overall Forced Labor in Country” used in the impact subcategory Forced Labor is derived using the Global Slavery Index. This source was classified with a score of 2 in five of the six quality criteria and with a score of 3 in the criterion of technological representativeness (the justification for this classification is presented in Supporting Information, Table S8). Hence, an uncertainty score of 2.17 was obtained based on the average of the six scores (see Supporting Information, Table S9), and consequently, a medium uncertainty level was defined (see Supporting Information, Table S9). According to Table S9 in the Supporting Information, 8 indicators were classified as having low uncertainty, 25 as having medium uncertainty, and 6 as having high uncertainty.

In contrast to the continuous scales that define the characterization factors used in environmental LCA studies, the SHI method employs a discrete scale with four levels: 1 (low risk), 2 (medium risk), 3 (high risk), and 4 (very high risk). To account for the uncertainty associated with these discrete risk levels, a probabilistic modeling approach based on Monte Carlo simulation was adopted, following the methodological rationale proposed by Santos et al. (2022).^18^ In this approach, uncertainty in characterization factors is represented using a uniform probability distribution, which is appropriate when empirical data are not available to define their probabilistic form.^40^ Considering that the SHI indicators are discrete and finite categories (levels 1–4), the uniform distribution was implemented in its discrete form, maintaining the principle of equiprobability and ensuring that only valid levels were sampled, with no intermediate values.

The use of uniform distributions is common in LCA studies, particularly in cases of epistemic uncertainty when only minimum and maximum limits are known and there is no information to describe the shape of uncertainty.^36,37,41–43^ Moreover, in contexts involving finite sets of mutually exclusive values, previous studies have demonstrated that discrete uniform distributions are suitable for preserving the integrity of categorical data and for avoiding artificial continuity.^44–46^

Accordingly, each indicator was assigned a discrete uniform probability distribution, allowing its risk level to vary symmetrically: ±1 point for low-uncertainty indicators (i.e., risk level variation of – 1, 0, + 1) with uniform probability of 1/3 (33%), ±2 points for medium-uncertainty indicators (i.e., risk level variation of – 2, – 1, 0, + 1, + 2) with uniform probability of 1/5 (20%), and ±3 points for high-uncertainty indicators (i.e., risk level variation of – 3 to +3) with uniform probability of 1/7 (14%). All variations were constrained within the SHI risk scale, ensuring that simulated values remained within the valid range from 1 (low risk) to 4 (very high risk).

At the extremes of the SHI scale (levels 1 and 4), variations are naturally limited by the bounds of the discrete classification; for example, indicators classified as low risk cannot assume values below level 1. Building on the principles outlined by André and Lopes (2012),^47^ Ewertowska et al. (2017),^48^ and Michiels and Geeraerd (2020),^49^ who argue that uncertainty can be represented within finite and realistic bounds, the stochastic modeling incorporated this boundary condition by applying the discrete uniform distribution exclusively to the valid categories within each uncertainty range, ensuring that all simulated values remained within the limits of the SHI scale. The variation levels and corresponding probabilities defined for each uncertainty class are summarized in Table 5.

To formalize the transition between risk levels under each uncertainty class, a discrete transition matrix was defined (Table 4). Each row represents the initial risk level *i*, and each column lists the feasible target levels after applying the symmetric variation ranges Δ ∈ { – 1,0, + 1}, { – 2, ···, + 2}, and { – 3, ···, + 3} for low, medium, and high uncertainty, respectively. Truncation at the boundaries of the SHI scale (1–4) ensures that all simulated outcomes remain within valid categories.

**Table 4:** Feasible Risk-Level Transitions for Each Uncertainty Class within the SHI Scale

	risk level			
uncertainty level	1 (low risk)	2 (medium risk)	3 (high risk)	4 (very high risk)
low uncertainty Δ ∈ {−1,0,1}	{1,2}	{1,2,3}	{2,3,4}	{3,4}
medium uncertainty Δ ∈ {−2, ···, + 2}	{1,2,3}	{1,2,3,4}	{1,2,3,4}	{2,3,4}
high uncertainty Δ ∈ {−3, ···, + 3}	{1,2,3,4}	{1,2,3,4}	{1,2,3,4}	{1,2,3,4}

For each starting risk level *i* and uncertainty class *u*, the feasible target set *S*(*i*, *u*) is given in Table 4, and probabilities are uniformly distributed among feasible outcomes according to eq 1.

P(j|i,u)={1|S(i,u)|ifj∈S(i,u)0,otherwise 1

This formulation preserves equiprobability among feasible outcomes while enforcing the discrete bounds of the SHI scale.^46–49^ Table 4 presents the corresponding uncertainty–risk matrix, showing all feasible transitions for each uncertainty class.

The variation levels and corresponding probabilities defined for each uncertainty class are summarized in Table 5. For example, according to Table 4, a social indicator with low uncertainty classified as low risk (1) can either remain at level 1 or move to level 2 in the Monte Carlo simulation.

The discrete probability distributions assigned to the risk level of each indicator were implemented by using the RiskDiscrete function in the @Risk add-in, referencing auxiliary tables that defined the allowed variation ranges and associated uniform probabilities for each uncertainty class. In this study, the indicators’ risk levels were randomly sampled from the defined probability distributions using the Latin Hypercube Sampling (LHS) technique, which provides higher accuracy than simple Monte Carlo sampling by ensuring a more stratified and representative coverage of the probability space.^50^ The simulation was executed until convergence, defined by the stabilization of the sample mean and variance of the simulated single scores and subcategory outcomes within a 3% threshold and a 95% confidence level, following the criterion proposed by Santos et al. (2022).^18^ Convergence was achieved after 6100 iterations, which were therefore adopted for all subsequent analyses.

**Table 5:** Risk Level Variation and Probability Assignment for Each Uncertainty Level

uncertainty level	risk level variation	probability
low	{−1, 0, 1}	33% each
medium	{−2, −1, 0, 1, 2}	20% each
high	{−3, −2, −1, 0, 1, 2, 3}	14% each

### Step 6: Analyze and Compare the Deterministic and Stochastic Results

The next step of the methodology consists of analyzing the deterministic and stochastic outputs (i.e., characterized results, normalized results, weighted results, or single scores) of the two previous steps. The lower the value of the potential social risk, the better it is from a social perspective. Since the goal of this study is to assess and compare the social performance of two components made with two material alternatives (Step 1), the analysis of the deterministic results involved comparing the single score of the ABS car dashboard with the single score of the cellulose-based car dashboard and the single score of the gypsum ship counter bar with the single score of the cellulose-based ship counter bar. This comparison allowed us to determine which material alternative is best from a social point of view for each of the two components analyzed. Moreover, the results of the most relevant impact subcategories of the ABS car dashboard and the gypsum ship counter bar were compared with the results of the same impact subcategories of the cellulose-based alternatives. This comparison allowed us to determine if the proposed cellulose-based alternatives contributed to the reduction of the most relevant social issues.

The stochastic results analyzed in this step should be the same as the deterministic results that were analyzed. However, analyzing stochastic results is more challenging because, unlike the deterministic impact assessment, where only a single estimate of each outcome is obtained (e.g., one single score for the ABS car dashboard), in the stochastic impact assessment, a range of each outcome is generated (e.g., 6100 single scores for the ABS car dashboard since 6100 iterations were performed). Statistical hypothesis testing has been used to analyze the outcomes of stochastic life cycle assessments.^51^ The objective of such tests is to evaluate whether the null hypothesis (*H*_0_) should be rejected, based on a comparison between a predefined significance level (α) and the calculated *p* value.^52^ If the *p* value is lower than or equal to the significance level, then the null hypothesis is rejected. In a comparative LCA, as is the case of the study presented in this article, the statistical hypothesis testing is used to determine if the true means of the potential social impacts obtained for the systems under analysis are equal (e.g., *H*_0_: μ_SS of ABS car dashboard_ = μ_SS of cellulose‑_ _based car dashboard_).^18^ To determine the most appropriate test for these pairwise comparisons, three assumptions must be assessed:^52^ independence of observations/variables, normality of data, and the homogeneity of variance. In a comparative LCA, the stochastic results are independent, as the outcomes for each system under analysis are not influenced by those of the other systems being analyzed. The assumption of normality of data can be waived when the sample size is sufficiently large (i.e., over 100 data points).^53^ Therefore, if the number of Monte Carlo iterations exceeds 100, as in the case of the study presented in this article, the assumption of normality is considered satisfied. However, if the number of iterations is fewer than 100, then normality should be verified using a test such as the Shapiro–Wilk test. Lastly, for the homogeneity of variance assumption, Levene’s test^54^ should be used to assess whether the variances of the stochastic results for the systems under analysis are equal. A *p* value less than or equal to the significance level indicates a lack of homogeneity in variance, while a *p* value greater than the significance level suggests that the assumption of equal variances holds. The pairwise comparisons previously mentioned (e.g., *H*_0_: μ_SS of ABS car dashboard_ = μ_SS of cellulose‑_ _based car dashboard_) can be tested using standard independent two samples *t* tests if the three assumptions are verified or a Welch’s *t* tests if the assumption of homogeneity of variances is not verified, for example.^18^ In this study, statistical hypothesis testing was performed in R,^55^ adopting a significance level of 0.05.^18^

The assumptions of the statistical tests were addressed and verified according to the methodological framework proposed by Santos et al. (2022).^18^ The independence of observations was ensured because the stochastic simulations for each system (conventional and cellulose-based) were performed separately with independent random sampling and no shared input data, so that the outcomes of one system did not influence the other. The normality assumption was considered satisfied given the large number of Monte Carlo iterations (*n* = 6100), which ensures, according to the Central Limit Theorem, that the sampling distribution of means follows a normal pattern. This behavior was visually confirmed through histograms showing symmetric and bell-shaped distributions for the stochastic results (Figure 3). The assumption of homogeneity of variances was verified using Levene’s test.^54^ In all cases, the equality of variances was not confirmed; therefore, Welch’s *t* test was applied in all pairwise comparisons, as it provides a reliable correction for unequal variances.^56^

**Figure 3:**
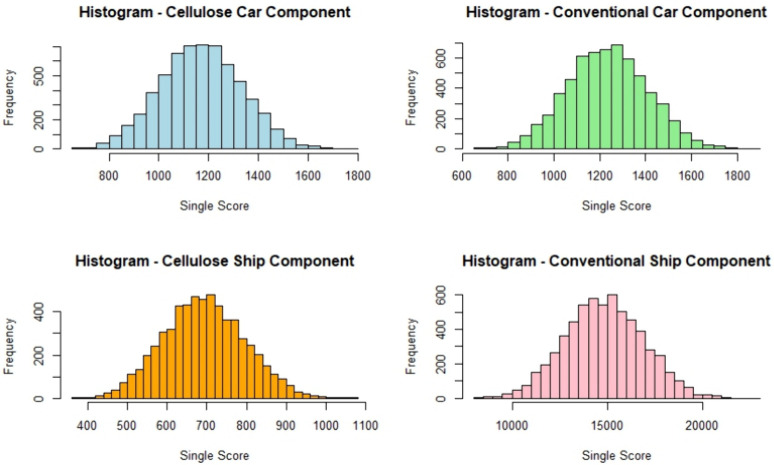
Histograms of stochastic single score distributions.

After analyzing the deterministic and stochastic results, the conclusions of both analysis should be compared. This comparison will allow determining how robust is the conclusion reached in the deterministic S-LCA that one system is better (or worse) than the other system(s) from a social perspective.^18^

The combination of deterministic and stochastic analyses in this study aimed to evaluate whether the implementation of stochastic modeling improves the interpretability and reliability of S-LCA results when compared to traditional deterministic assessments. Conducting both analyses allowed verification of whether incorporating uncertainty provides additional insights rather than assuming this a priori in order to support greater confidence among decision-makers. In this context, the deterministic analysis served as a baseline, representing the conventional S-LCA approach, while the stochastic analysis introduced uncertainty into the same model to assess how the conclusions changed or remained stable. This comparison enabled validation of whether the probabilistic approach yielded consistent results with the deterministic one and provided a basis for demonstrating the added value of stochastic modeling in S-LCA.

A practitioner or researcher who intends to perform only an uncertainty-based (stochastic) analysis could skip Step 4 (deterministic assessment) and Step 6 (comparison between deterministic and stochastic results), as these steps were specifically included to validate and demonstrate the added value of stochastic modeling in this research. For this study, however, maintaining both approaches was essential to ensure that the proposed methodology is not only theoretically consistent but also empirically justified in terms of its usefulness for S-LCA practice.

## 3 Results

### 3.1 Deterministic Results

The deterministic results obtained for the four systems under analysis are presented in Figures 4 and 5. For the car dashboard, the cellulose-based alternative had a higher single score (1,018.92 Pt) than the conventional ABS system (579.52 Pt), suggesting a lower social risk in the conventional option.

**Figure 4:**
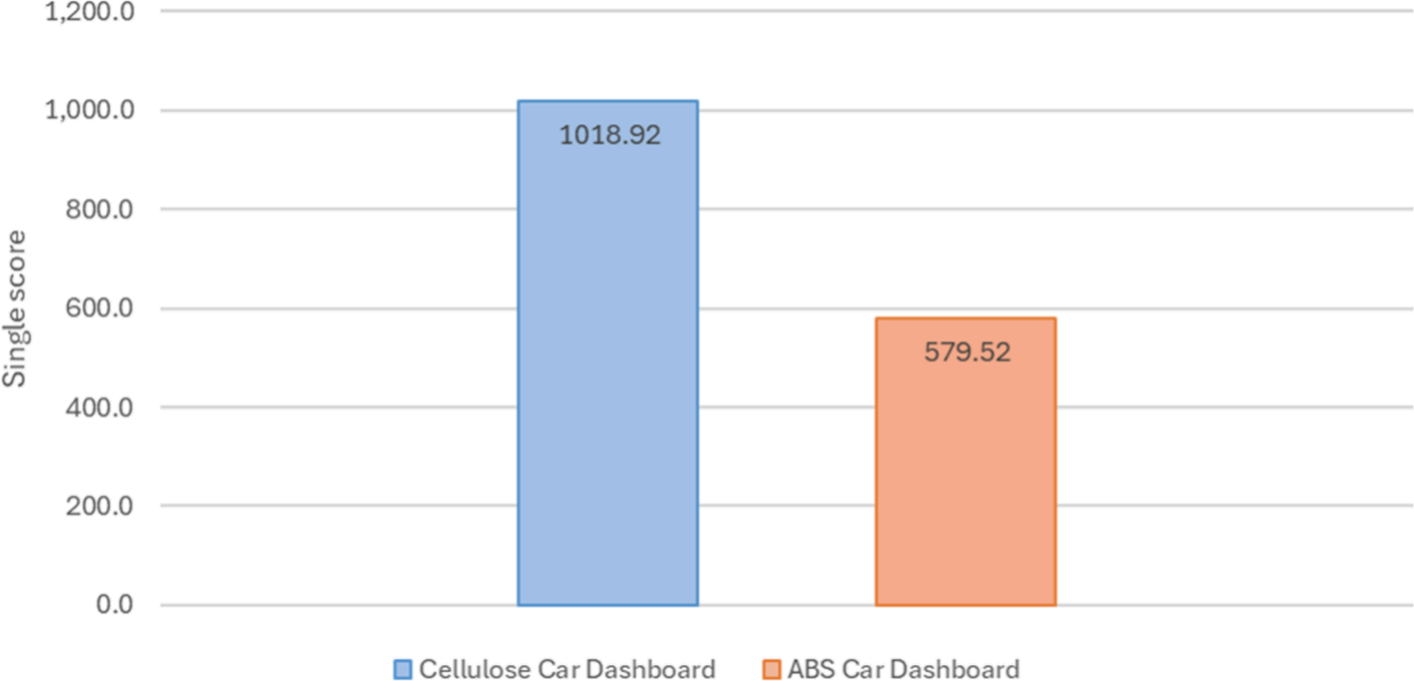
Deterministic single scores for the car dashboard (ABS vs cellulose-based).

**Figure 5:**
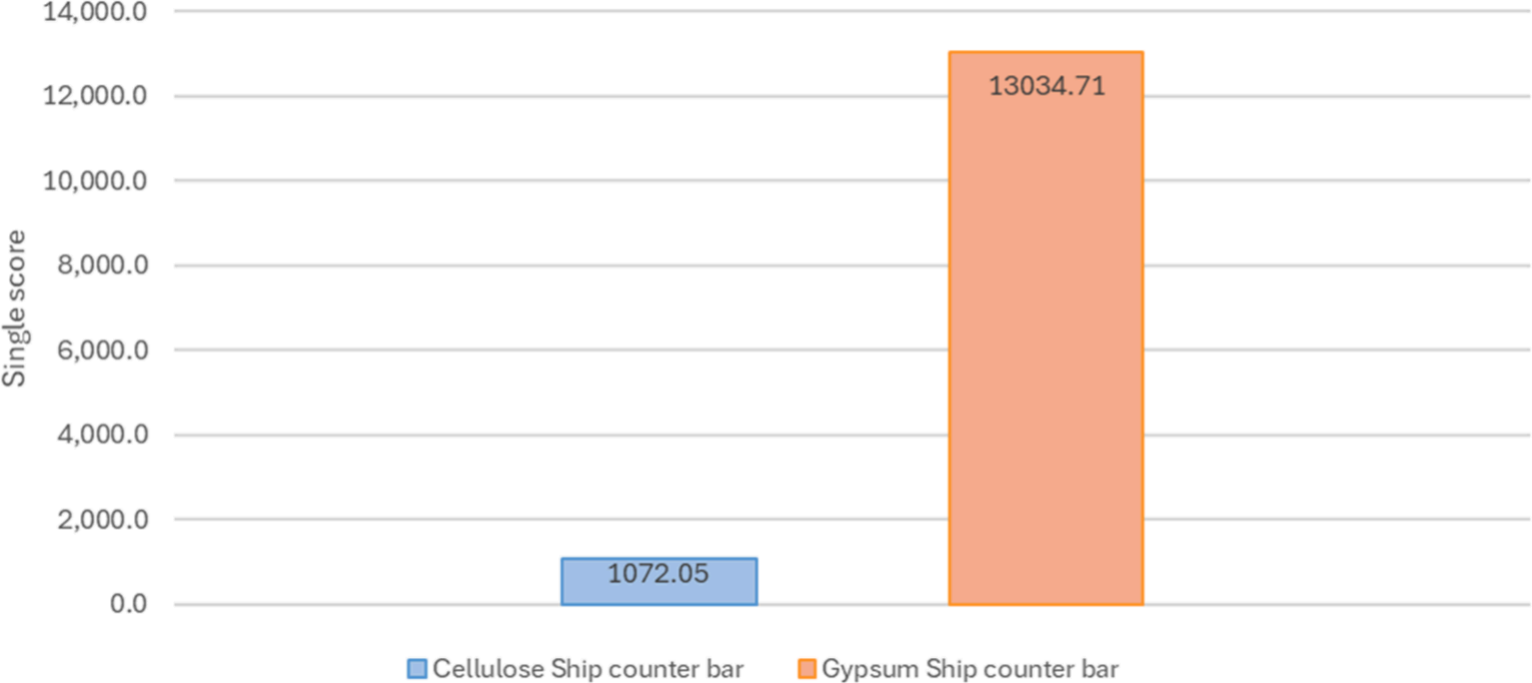
Deterministic single scores for the ship counter bar (gypsum vs cellulose-based).

On the other hand, for the ship counter bar, the alternative made with the cellulose-based material presented a lower overall potential social impact (1,072.05 Pt) compared to the conventional system using gypsum (13,034.71 Pt), indicating a better social performance.

This study also included a disaggregated analysis of the deterministic results by social impact subcategories, as shown in Figures 6 and 7. This breakdown provides greater insight into which specific issues contribute the most significantly to the overall social performance of each component.

**Figure 6:**
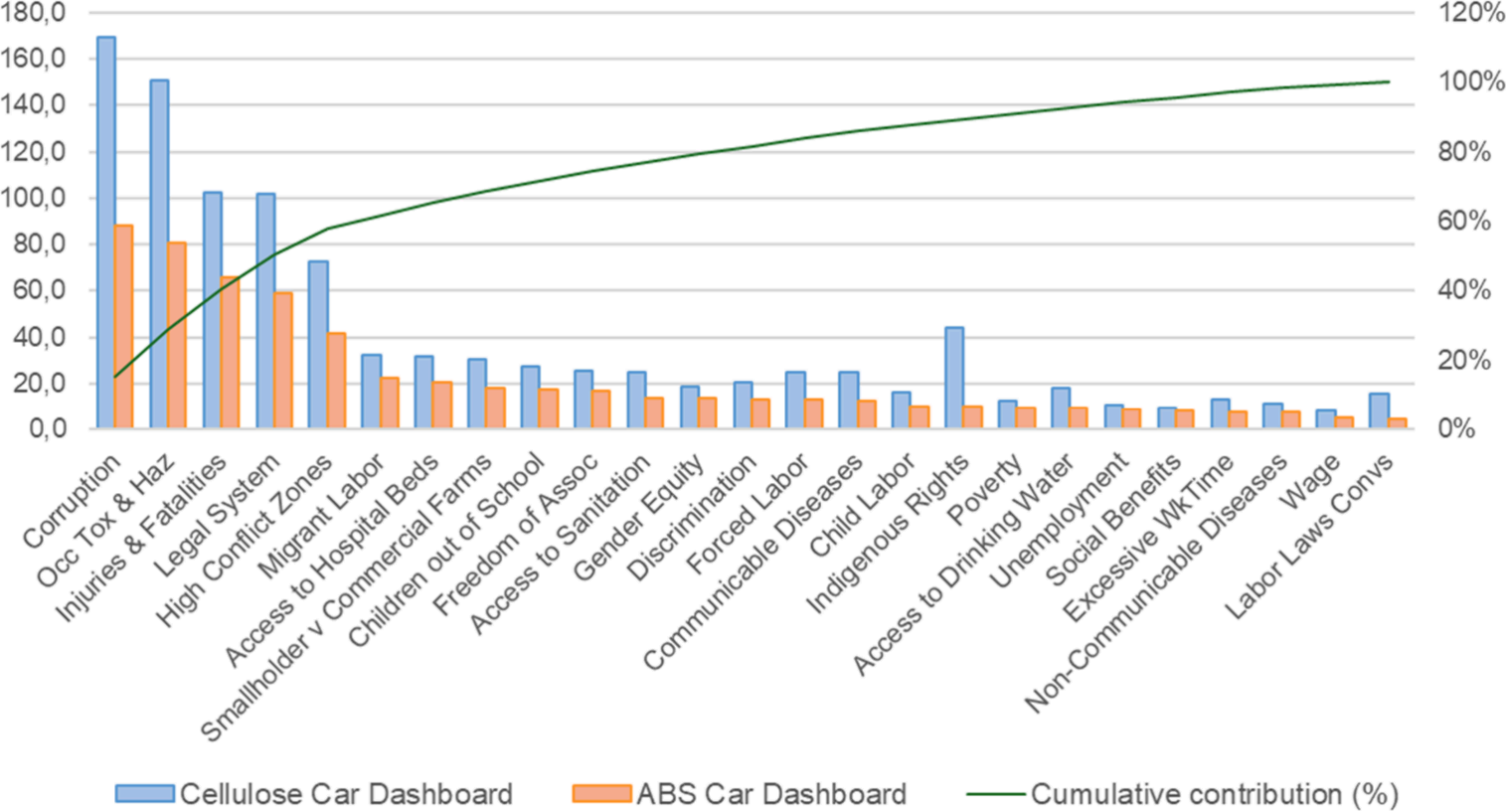
Deterministic social impacts by subcategory – car dashboard (ABS vs cellulose-based).

**Figure 7:**
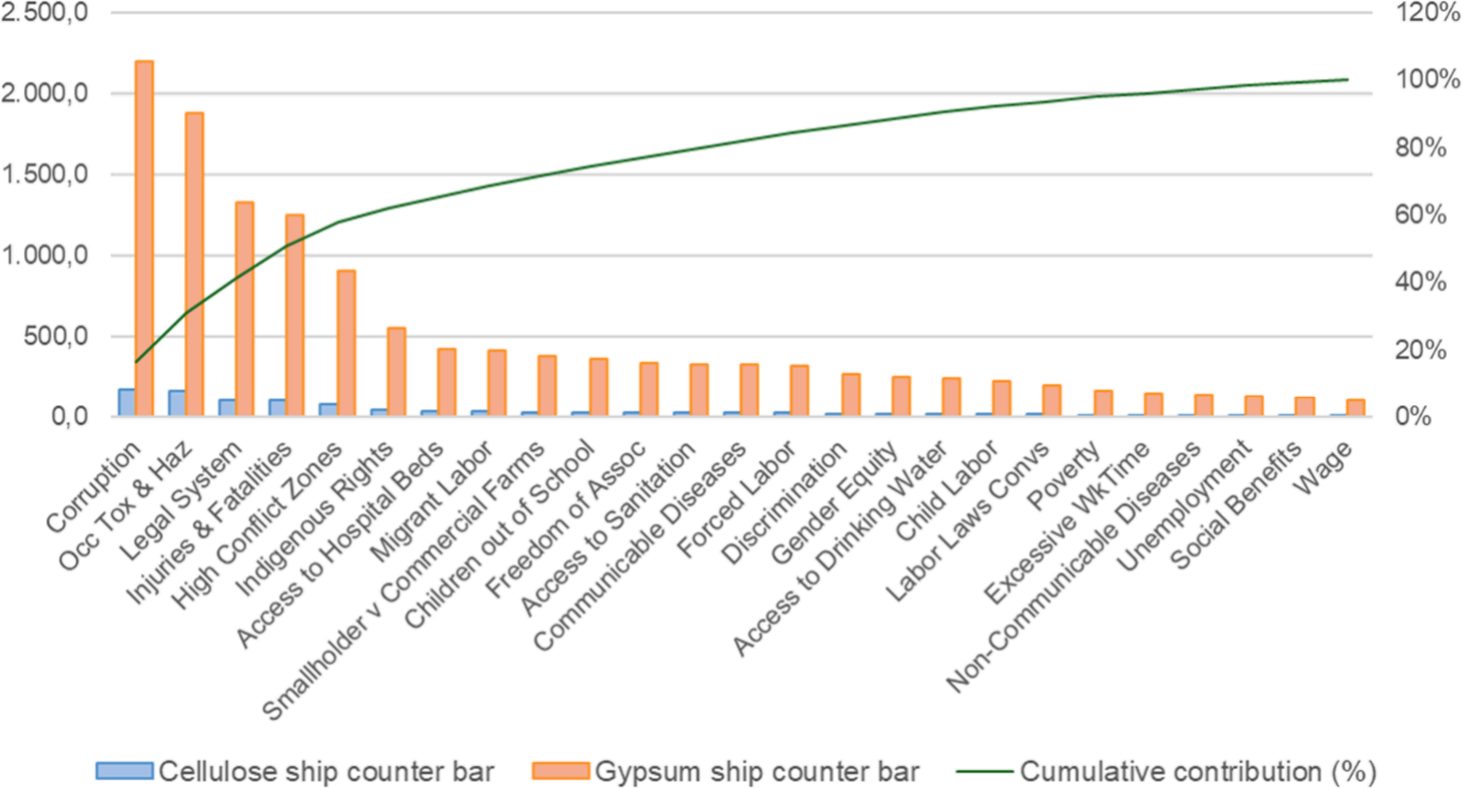
Deterministic social impacts by subcategory – ship counter bar (gypsum vs cellulose-based).

To determine which subcategories were most relevant, a ranking was performed based on the normalized results of the conventional alternatives (ABS and gypsum). This approach was chosen to highlight the areas where the current materials pose the most significant social risks and to assess whether the cellulose-based alternative could help reduce those impacts. By using the conventional systems as a reference point, the analysis focuses on the potential of the novel material to address the most critical social challenges currently present. Moreover, following the Pareto principle, which states that a small number of causes are responsible for most effects, the five subcategories with the highest contribution to the total social impact were selected for further analysis. This corresponds to 20% of the 25 subcategories assessed, allowing the study to concentrate on the most influential factors in a simplified and effective manner.

For the car dashboard, the cellulose-based alternative shows higher impact values across all subcategories when compared with the conventional ABS system. The five subcategories with the highest overall impact, ranked according to the normalized values of the conventional alternative, are (i) Corruption; (ii) Occupational Toxics and Hazards; (iii) Injuries and Fatalities (iv) Legal System; and (v) High Conflict Zones

These five subcategories represent the most significant contributions to the social impact in both systems, indicating that these issues are consistently associated with higher social risks across multiple dimensions, particularly in terms of governance and worker safety.

In contrast, for the ship counter bar, the conventional alternative made from gypsum shows significantly higher impacts in all subcategories when compared with the cellulose-based version. The five subcategories with the highest overall impact are the same as those identified for the car dashboard, with slight differences in their ranking: (i) Corruption; (ii) Occupational Toxics and Hazards; (iii) Legal System; (iv) Injuries and Fatalities; and (v) High Conflict Zones.

In this case, the cellulose-based alternative results in markedly lower social impacts, pointing to better overall social performance. These findings illustrate how the social risks vary depending not only on the material used but also on the context and application, especially due to differences in supply chain configurations and geographical sourcing.

The selection of the five subcategories with the highest impact (20% of the 25 subcategories assessed) was guided by the Pareto principle, aiming to focus on the most influential factors in a simplified and effective manner.^57^ This choice reflects the empirical contribution analysis performed on the normalized deterministic results, which showed a clear concentration of impacts in a small subset of subcategories. The contribution analyses (Figures 5 and 6) indicate that these five categories cumulatively represent approximately 60% of the total contribution of potential social risk in conventional systems. Although this value does not reach the empirical 80/20 proportion, it represents the most relevant and materially influential share of the social risk. Focusing the analysis on this portion allows for an in-depth discussion of the most critical dimensions of social risk, such as governance and occupational health and safety. Including a larger number of categories (approximately 12, to reach 80% contribution) would add factors of low individual magnitude, thereby diluting the analytical focus and reducing the interpretability and transparency of the contribution analysis.^58,59^

In methodological terms, the contribution analysis based on normalized results was the most appropriate and consistent option for identifying dominant subcategories since the alternative approaches such as threshold sensitivity methods require continuous parametric models and defined quantitative thresholds for input variables,^36,60,61^ whereas the SHI subcategories are discrete and qualitative risk levels with no parametric structure that would allow threshold variation analysis. Similarly, global sensitivity techniques are designed for models with quantitative inventories or numerical parameters,^11^ which is not the case for social risk classifications in the SHI.

A more detailed examination of the deterministic results reveals that these differences arise from the specific sectors, processes, and regions represented in each system as well as from the social indicators driving their results. To make these patterns explicit, Figures 8 and 9 present the process contribution analysis for the five dominant subcategories in each product system.

**Figure 8:**
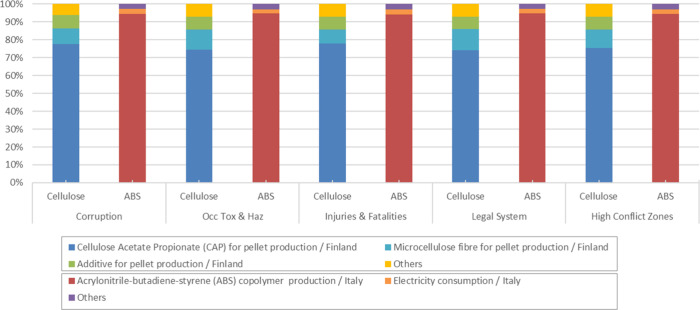
Process contribution analysis: car dashboard systems.

**Figure 9:**
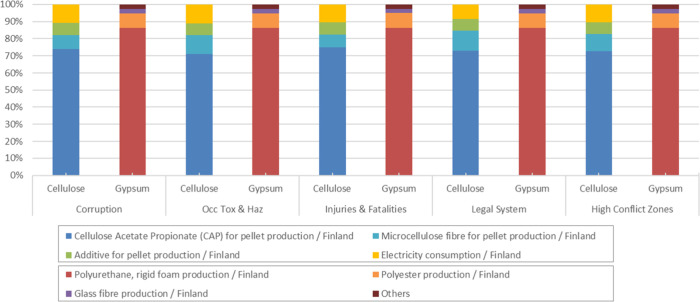
Process contribution analysis: Ship counter bar systems.

For the car dashboard, Figure 8 shows that the cellulose-based system is dominated by the production of cellulose-based pellets in Finland, which concentrates most worker hours and is linked to the chemical and forestry sectors. In the SHDB, these sector-region combinations show high risks for occupational toxics and hazards and medium to high risks for occupational injuries. Indirect risk transmission through trade associated with the Finnish chemical sector also contributes to the relevance of corruption and the legal system as influential subcategories. In contrast, the ABS system is dominated by chemical production in Italy, which corresponds to a sector-region combination with lower risks in the most influential subcategories according to the SHDB. As a result, the total impacts for the ABS dashboard are lower.

For the ship counter bar, the gap between the alternatives is much larger. The gypsum-based system involves much higher volumes of worker hours in the dominant processes (polyurethane, polyester, and glass fiber), as shown by the relative contributions in Figure 9. Because the SHDB calculates the potential risk by multiplying the risk intensity by the number of worker hours, the overall impact increases proportionally. Thus, even if the unit level risk is comparable to that of the sectors in the cellulose-based system, the significantly larger volume of worker hours leads to a much higher total impact.

In the cellulose-based system, most worker hours are concentrated in CAP and microcellulose fiber production, which belong to sectors with elevated risks in the subcategories occupational toxics and hazards and occupational injuries. However, because the aggregated risk results from the combination of sector-level intensity and the distribution of worker hours, the final risk profile does not reach the levels observed in the gypsum system, which brings together multiple high intensity sectors simultaneously.

### 3.2 Stochastic Results

While the deterministic results provide valuable insights into the social performance of each system, they do not capture the uncertainty associated with the CFs used in the calculation of single scores. To address this limitation, a new methodology was employed. For each of the four components under study, 6100 iterations were performed, generating probability distributions of single score values that reflect the variability introduced by uncertainty in the input parameters, particularly the social CFs.

As described in Step 6 of the methodology section, three assumptions were considered before applying statistical tests to these stochastic results: (i) independence of observations, (ii) normality, and (iii) homogeneity of variance. The first assumption was met by considering that the results of each component were independent of one another. The second was assumed to be based on the Central Limit Theorem due to the use of 6100 iterations. To verify the third assumption, Levene’s test was applied to assess whether the variances between the two groups compared were equal. This test checks whether the variability of the data is similar across groups, which is essential for determining whether a standard *t* test or Welch’s *t* test should be used. The results, shown in Table 6, indicated *p* values below the 0.05 significance level for both comparisons, suggesting unequal variances and justifying the use of Welch’s *t* test. This test assesses whether the means of two groups differ significantly, even when their variances are not equal.

**Table 6:** Levene’s Test for Homogeneity of Variances and Selected Statistical Test

comparison	degrees of freedom (df)	test statistic (Levene’s *F*)	*p* value	selected test
car dashboard (ABS vs cellulose)	1	1,127.78	1.47 × 10^–236^	Welch
ship counter bar (gypsum vs cellulose)	1	9,183.91	0.00	Welch

The results of Welch’s *t* tests are presented in Table 7. For both the car dashboard and the ship counter bar, the *p* values obtained were well below 0.05, confirming that the differences in mean social impact between the conventional and cellulose-based options are statistically significant. These results reinforce the conclusions drawn from the deterministic analysis.

**Table 7:** Welch’s *t*-Test Results for the Stochastic Single Scores

comparison	degrees of freedom (df)	test statistic (*t*)	*p* value	mean (conv)	mean (cell)	CI lower	CI upper	test conclusion
car dashboard (ABS vs cellulose)	10,224.72	–191.98	0.00	693.04	1,169.52	–481.35	–471.62	different
ship counter bar (gypsum vs cellulose)	6,188.46	514.70	0.00	14,826.79	1,234.70	13,540.33	13,643.86	different

The distributions obtained through the simulations are visualized in Figures 10 and 11. For the car dashboard, the cellulose-based alternative showed a consistently higher potential social impact than the ABS version, with minimal overlap between distributions. The mean single score for the cellulose-based dashboard was 1,169.52 Pt, compared to 693.04 Pt for the ABS version, resulting in a difference of approximately 476 Pt. Statistical analysis using Welch’s *t* test confirmed that this difference is significant (*p* value <0.001), meaning it is highly unlikely to result from random variation. The 95% confidence interval for the difference in means (from −481.35 to −471.62 Pt) does not include zero, confirming that the cellulose-based alternative consistently presents higher potential social impacts than the conventional ABS system.

**Figure 10:**
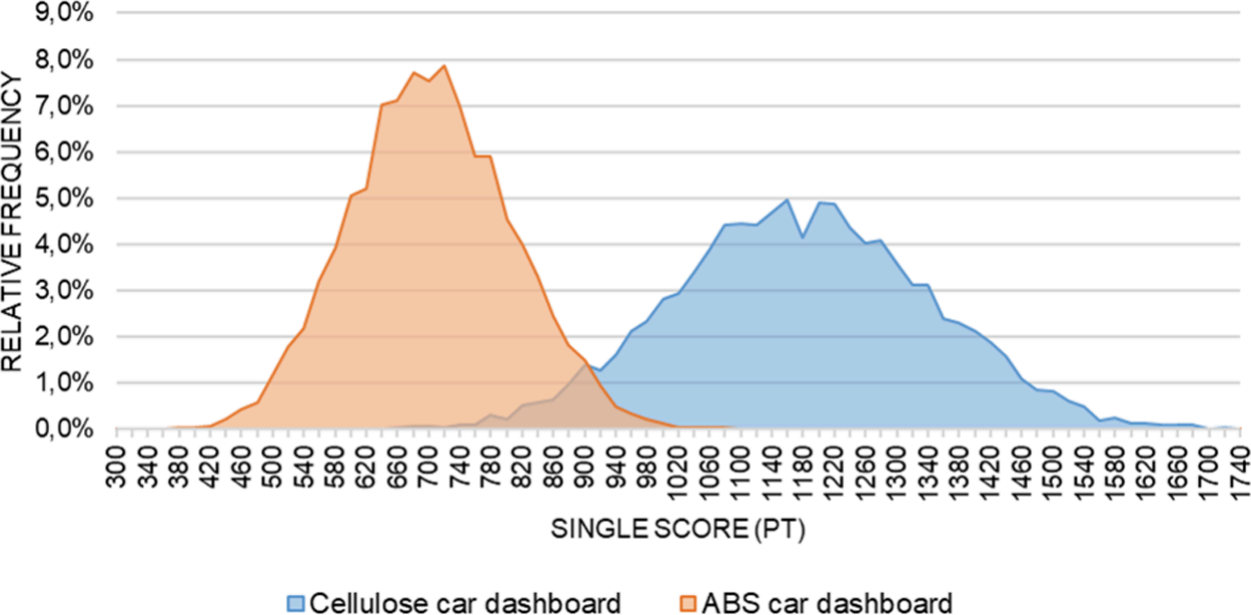
Distribution of stochastic single scores for the car dashboard (ABS vs cellulose-based).

**Figure 11:**
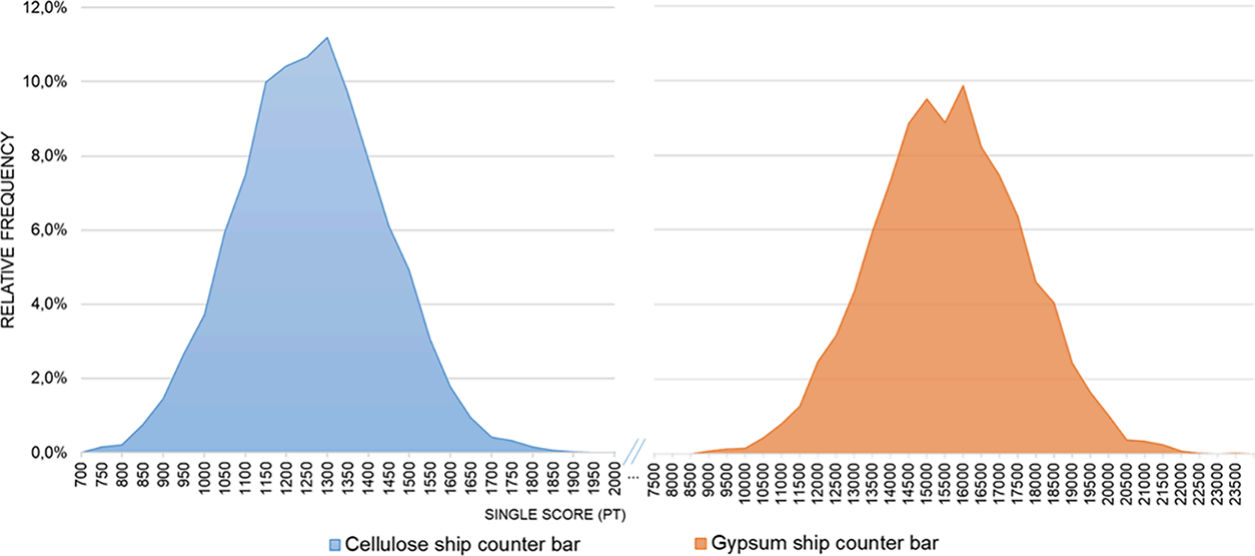
Distribution of stochastic single scores for the ship counter bar (gypsum vs cellulose-based).

For the car dashboard, the higher stochastic values of the cellulose-based alternative are consistent with the deterministic process contribution analysis. As shown in Figure 8, this system is dominated by cellulose pellet production in Finnish chemical and forestry sectors that present higher SHDB risk levels in the dominant subcategories and concentrate most worker hours. In the stochastic results, this structure appears as a distribution shifted toward higher single score values, indicating that the difference between materials arises from these sector and region risk patterns rather than from the statistical procedure itself.

In contrast, for the ship counter bar, the cellulose-based alternative demonstrated markedly lower social impact values compared to the gypsum system, also with no distribution overlap. The mean single score for the gypsum system was 14,826.79 Pt, while the cellulose-based version averaged 1,234.70 Pt, a substantial difference of 13,592 Pt. This difference is also statistically significant (*p* value <0,001), with a 95% confidence interval from 13,540.33 to 13,643.86 Pt, confirming that the cellulose-based material performs better in this case.

Since the magnitude of the simulated values differs widely between the two materials in the ship counter bar, with cellulose-based scores concentrated near 1,200 Pt and gypsum-based scores near 14,800 Pt, the *x*-axis of Figure 11 was adapted to appropriately represent the range of each distribution and ensure clear visualization. These graphical results confirm the statistical findings and illustrate the magnitude and direction of the differences under uncertainty.

For the ship counter bar, the much lower stochastic values of the cellulose-based alternative reflect the worker hour and sector patterns described in the deterministic results. As shown in Figure 9, the gypsum system aggregates high volumes of worker hours in polyurethane, polyester, and glass fiber processes, which the SHDB classifies with higher risk levels in the dominant subcategories. In contrast, the cellulose-based system is mainly influenced by CAP and microcellulose fiber production, which have elevated risks but do not combine as many high intensity processes as the gypsum system. The clear separation between the distributions in Figure 11 shows that this difference in sector and process compositions persists when uncertainty in social CFs is taken into account.

In addition to the comparison of single scores, a stochastic analysis was conducted for the five social impact subcategories that showed the highest contribution in the deterministic assessment of the conventional alternatives. These subcategories (Corruption, Occupational Toxics and Hazards, Injuries and Fatalities, Legal System, and High Conflict Zones) were selected based on their relevance in the normalized results of the ABS car dashboard and the gypsum ship counter bar, as previously described in the deterministic analysis. It is important to note that the objective of the stochastic analysis by subcategory is not to reassess which categories are the most impactful but rather to verify whether the differences observed between the conventional materials and the cellulose-based alternative remain consistent under data uncertainty.

The same approach applied to the single score was used for each subcategory: a Monte Carlo simulation with 6100 iterations using discrete probability distributions based on the uncertainty level of each indicator. The simulations generated probability distributions of impact values, reflecting the variation introduced by uncertainty in the social CFs. Levene’s test was then applied to assess the homogeneity of variances between the technologies in each of the five subcategories. The results showed *p* values below the significance level of 0.05 in all cases, indicating that the variances between the groups (conventional materials and cellulose-based materials) cannot be considered homogeneous. The results of Levene’s test are presented in Table 8. Accordingly, Welch’s *t* test was adopted for the subsequent analyses, as it is more appropriate in situations with unequal variances.

**Table 8:** Levene’s Test Results for the Social Subcategories

	Comparison	Degrees of Freedom (df)	Test Statistic (Levene’s F)	*p* value	Selected Test
car dashboard (ABS vs cellulose)	Corruption	1	1,303.91	2.30 × 10^–271^	Welch
	Occupational Toxics and Hazards	1	1,998.93	0.00	Welch
	Injuries and Fatalities	1	2,155.42	0.00	Welch
	Legal System	1	817.21	4.96 × 10^–174^	Welch
	High Conflict Zones	1	4,073.10	0.00	Welch
ship counter bar (gypsum vs cellulose)	Corruption	1	9,840.60	0.00	Welch
	Occupational Toxics and Hazards	1	10,046.99	0.00	Welch
	Legal System	1	9,835.76	0.00	Welch
	Injuries & Fatalities	1	16,384.45	0.00	Welch
	High Conflict Zones	1	24,272.48	0.00	Welch

The complete results of Welch’s *t* test are presented in Table 9. For the car dashboard, the conventional ABS alternative showed lower mean social impact values in all subcategories when compared with the cellulose-based material. This reinforces the findings from both deterministic and stochastic analyses observed in the aggregated scores. In contrast, for the ship counter bar, the mean social impact values were significantly lower for the cellulose-based alternative in all analyzed subcategories, once again indicating better social performance of the new technology.

**Table 9:** Welch’s *t*-Test Results for the Social Subcategories

	comparison	degrees of freedom (df)	Test Statistic (*t*)	*p* value	mean (conv)	mean (cell)	CI lower	CI upper	test conclusion
car dashboard (ABS vs cellulose)	Corruption	10,063.44	–63.01	0.00	10.23	18.12	–8.13	–7.64	different
	Occupational Toxics and Hazards	9,385.87	–82.69	0.00	9.46	16.89	–7.61	–7.25	different
	Injuries and Fatalities	9,185.03	–69.56	0.00	7.28	12.66	–5.54	–5.23	different
	Legal System	11,489.21	–36.02	0.00	8.60	12.85	–4.49	–4.02	different
	High Conflict Zones	9,685.11	–41.78	0.00	11.34	19.43	–8.46	–7.70	different
ship counter bar (gypsum vs cellulose)	Corruption	6,189.95	159.18	0.00	230.50	19.15	208.74	213.95	different
	Occupational Toxics and Hazards	6,193.71	198.98	0.00	211.50	18.06	191.53	195.34	different
	Legal System	6,183.39	126.22	0.00	164.36	13.43	148.59	153.27	different
	Injuries & Fatalities	6,196.20	167.38	0.00	155.24	13.55	140.03	143.35	different
	High Conflict Zones	6,188.06	106.37	0.00	244.11	20.64	219.35	227.59	different

The results of Welch’s *t* tests confirmed statistically significant differences between the conventional materials and the cellulose-based alternative across all five subcategories analyzed, for both the car dashboard and the ship counter bar. The *p* values were below the 0.05 significance level in all comparisons, and the confidence intervals did not include zero, confirming that the differences are significant and clearly showing which material has higher social impacts.

In the case of the car dashboard (ABS vs cellulose), the results indicated that the cellulose-based material showed significantly higher mean values across all analyzed subcategories (Corruption, Occupational Toxics and Hazards, Injuries and Fatalities, Legal System, and High Conflict Zones), pointing to a less favorable social performance of this alternative compared to the conventional material.

For the ship counter bar (gypsum vs cellulose), the results were the opposite: the cellulose-based alternative presented significantly lower mean values in all subcategories, indicating superior social performance compared to the conventional system. These findings reinforce the conclusions from the deterministic analysis, confirming that the differences between the materials remain consistent, even when accounting for the uncertainty associated with input data.

Partial overlap between distributions does not imply that the materials perform similarly. Across all subcategories, the cellulose-based alternative shows a systematic shift in the central tendency toward higher values in the car dashboard and toward lower values in the ship counter bar. This means that the relative performance remains consistent, even when the distributions share common ranges. For instance, in the car dashboard Corruption subcategory, both materials share a common range between approximately 10 and 20 Pt. However, the cellulose-based alternative shows higher values in most of the simulated outcomes. Its median and interquartile range lie entirely above those of ABS, meaning that although some simulated values coincide, the typical results remain different. The rightward extension of the cellulose distribution reflects this pattern and explains how overlap can occur even when one material consistently performs worse.

The probability distributions of the simulated social impact values for each subcategory are presented in Figures 12 and 21. For the car dashboard, the simulated social impact values are consistently higher for the cellulose-based alternative, with little overlap between the distributions. The same pattern, but in the opposite direction, is observed for the ship counter bar, where the impacts of the conventional technology clearly exceed those of the cellulose-based alternative. These visual results reinforce the conclusions of the statistical tests and demonstrate the magnitude of the differences under uncertainty.

**Figure 12:**
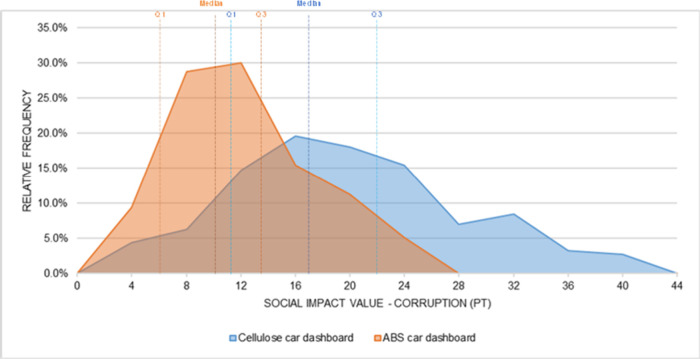
Probability distribution: Corruption (car dashboard).

Figures 12 and 16 show the distributions of the simulated values for the five social impact subcategories analyzed in the car dashboard. In all of them, the values associated with the cellulose-based material were consistently higher than those of the conventional ABS material, reflecting the trend identified in the statistical tests.

In the Corruption subcategory (Figure 12), the distributions for ABS and cellulose show partial overlap, especially in the range between 10 and 20 Pt. However, the central tendency of the cellulose distribution is shifted toward higher values. The median and the first (Q1) and third quartiles (Q3) of the cellulose-based alternative are all higher than those of ABS, indicating that in most simulated outcomes, the cellulose option presents greater social impact values. The rightward extension of the cellulose distribution reflects the presence of simulations with higher impact values and is consistent with this shift in the central tendency. Welch’s *t* test confirmed that the difference is statistically significant at the 95% confidence level, with a confidence interval for the difference in means ranging from −8.13 to −7.64 Pt, which does not include zero. Additional descriptive statistics supporting this interpretation are provided in Table S10 in the Supporting Information.

A similar pattern is observed in the Occupational Toxics and Hazards subcategory (Figure 13), and the distributions also show partial overlap, but the cellulose-based alternative is consistently shifted toward higher values. The median and the quartile values (Q1 and Q3) (Table S10 in the Supporting Information) for the cellulose system are higher than those of ABS, indicating that in most simulated outcomes, the cellulose alternative presents greater social impact values. The wider spread of the cellulose distribution reflects the range of values generated when uncertainty in the social CFs is applied to the sector and region combinations that dominate this system. Statistical analysis using Welch’s *t* test confirmed a significant difference between the groups at the 95% confidence level. The resulting confidence interval for the difference in means spans from −7.61 to −7.25 Pt and remains entirely below zero, reinforcing the conclusion that the cellulose-based material has consistently higher impact values in this subcategory.

**Figure 13:**
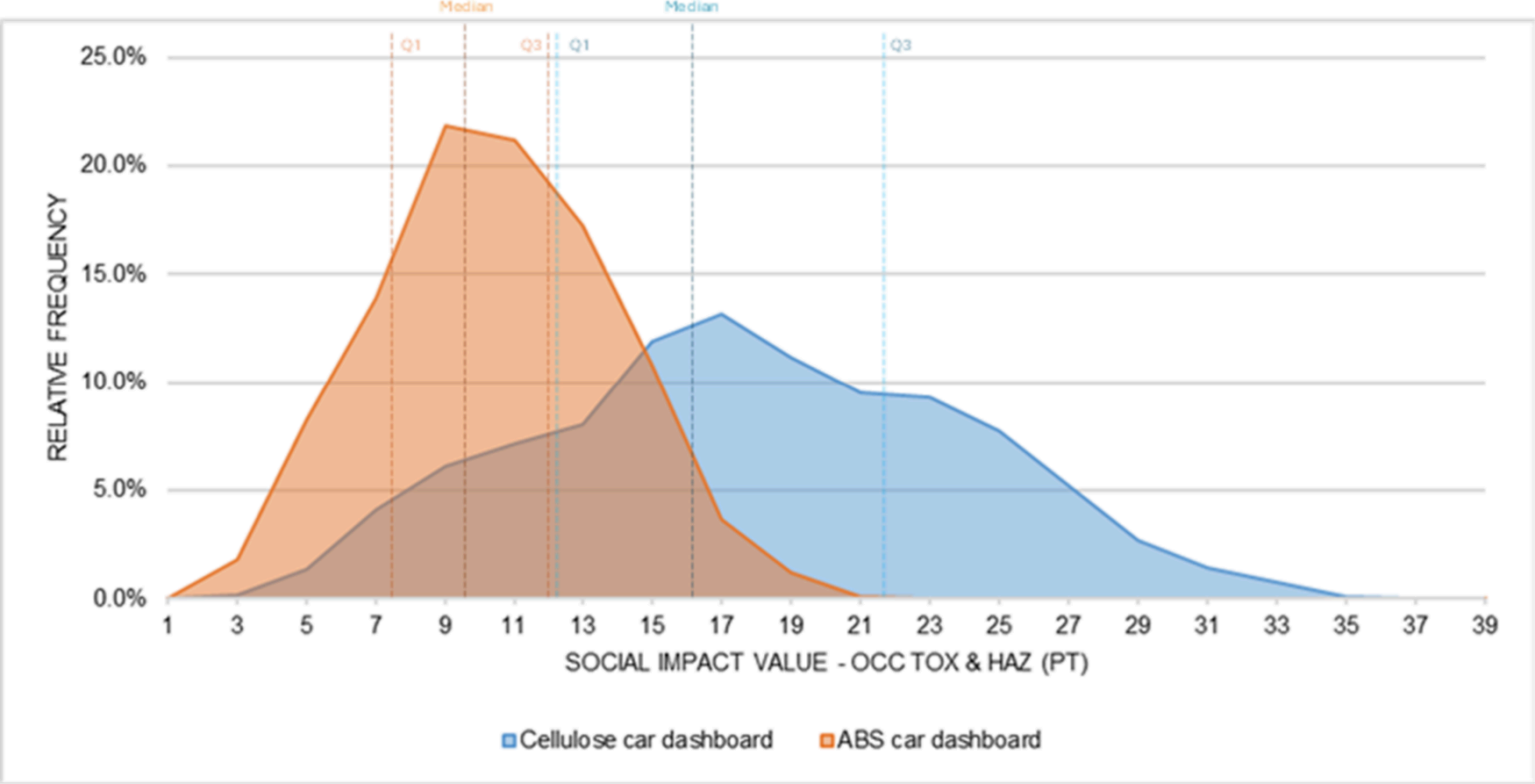
Probability distribution: Occupational Toxics and Hazards (car dashboard).

In the Injuries and Fatalities subcategory (Figure 14), the cellulose curve is more dispersed and shifted slightly to the right compared to the ABS curve, indicating higher average impact values. This pattern is consistent with the deterministic results in which the cellulose system already presented higher contributions in this subcategory. When uncertainty is introduced, the simulated values vary around these underlying contributions and the relative position of the two materials remains unchanged. Although the curves overlap, the location of their central values shows that the cellulose system yields higher outcomes in most iterations, which is consistent with the statistical test results. The Welch’s *t* test confirmed that this difference is statistically significant, with a 95% confidence interval ranging from −5.54 to −5.23 Pt. Since the interval lies entirely below zero, it supports the conclusion that the cellulose-based material systematically presents greater social impacts in this subcategory.

**Figure 14:**
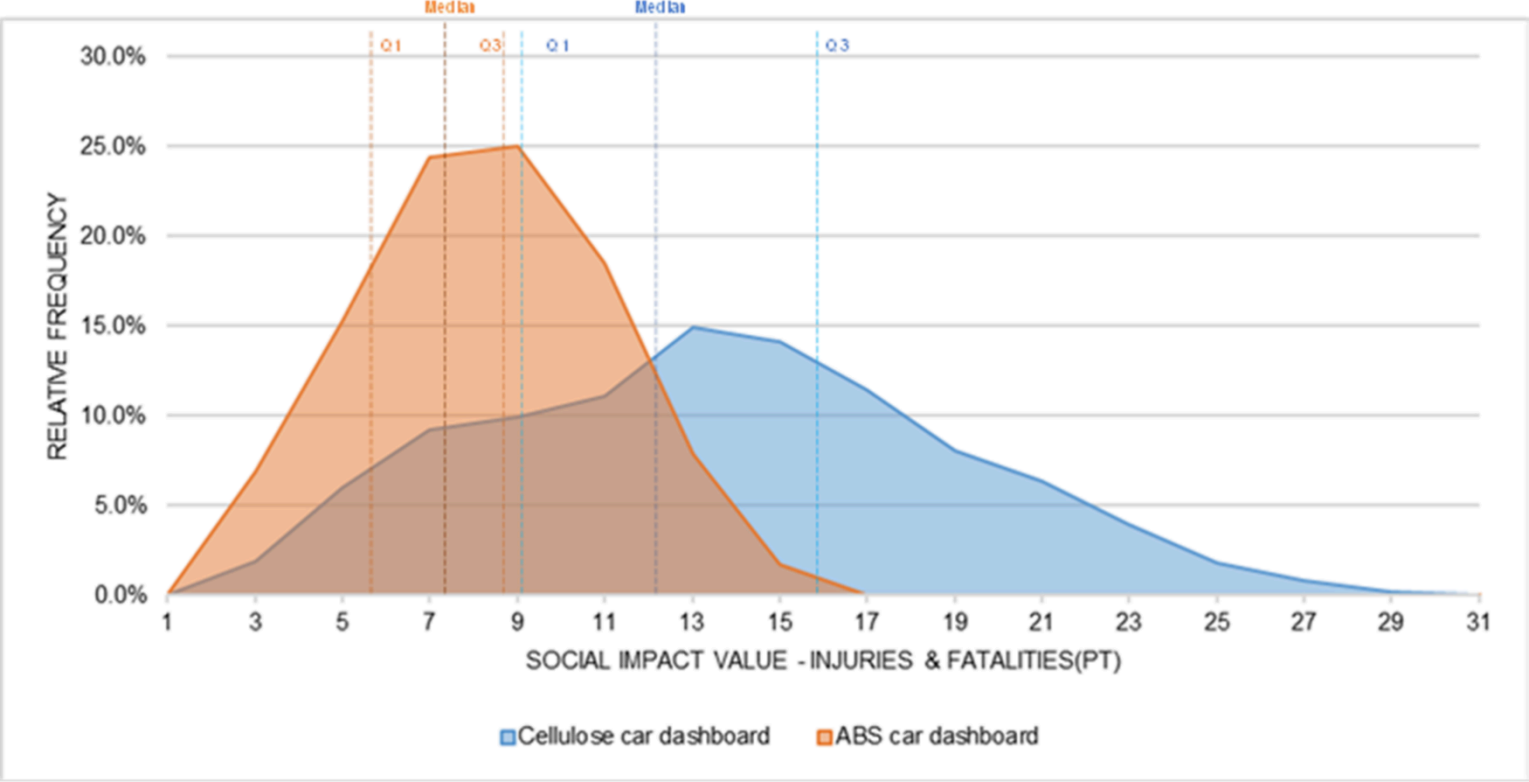
Probability distribution: Injuries and Fatalities (car dashboard).

In the Legal System subcategory (Figure 15), the distributions for cellulose and ABS show substantial overlap (particularly between 5 and 20 Pt), but the central values of the cellulose distribution remain higher across the simulated range. The cellulose curve also extends further to the right, which aligns with the higher deterministic contributions observed for this subcategory. Even within the overlapping region, most simulated outcomes for the cellulose system fall above those of the ABS, leading to a higher mean value. Welch’s *t* test confirmed this difference as statistically significant, with a 95% confidence interval for the difference in means ranging from −4.49 to −4.02 Pt. As this interval does not include zero, it indicates a robust distinction between the two materials in this impact category.

**Figure 15:**
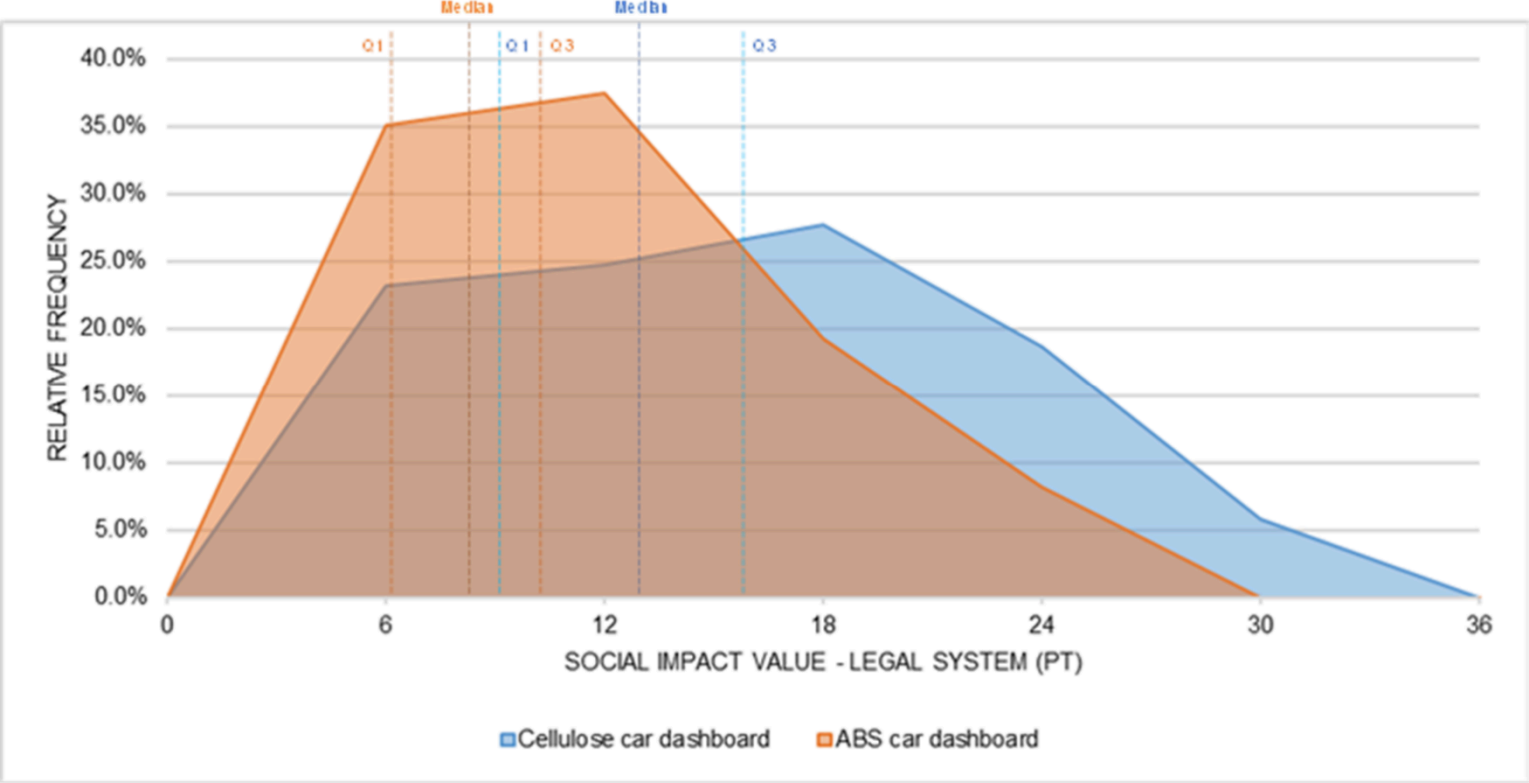
Probability distribution: Legal System (car dashboard).

**Figure 16:**
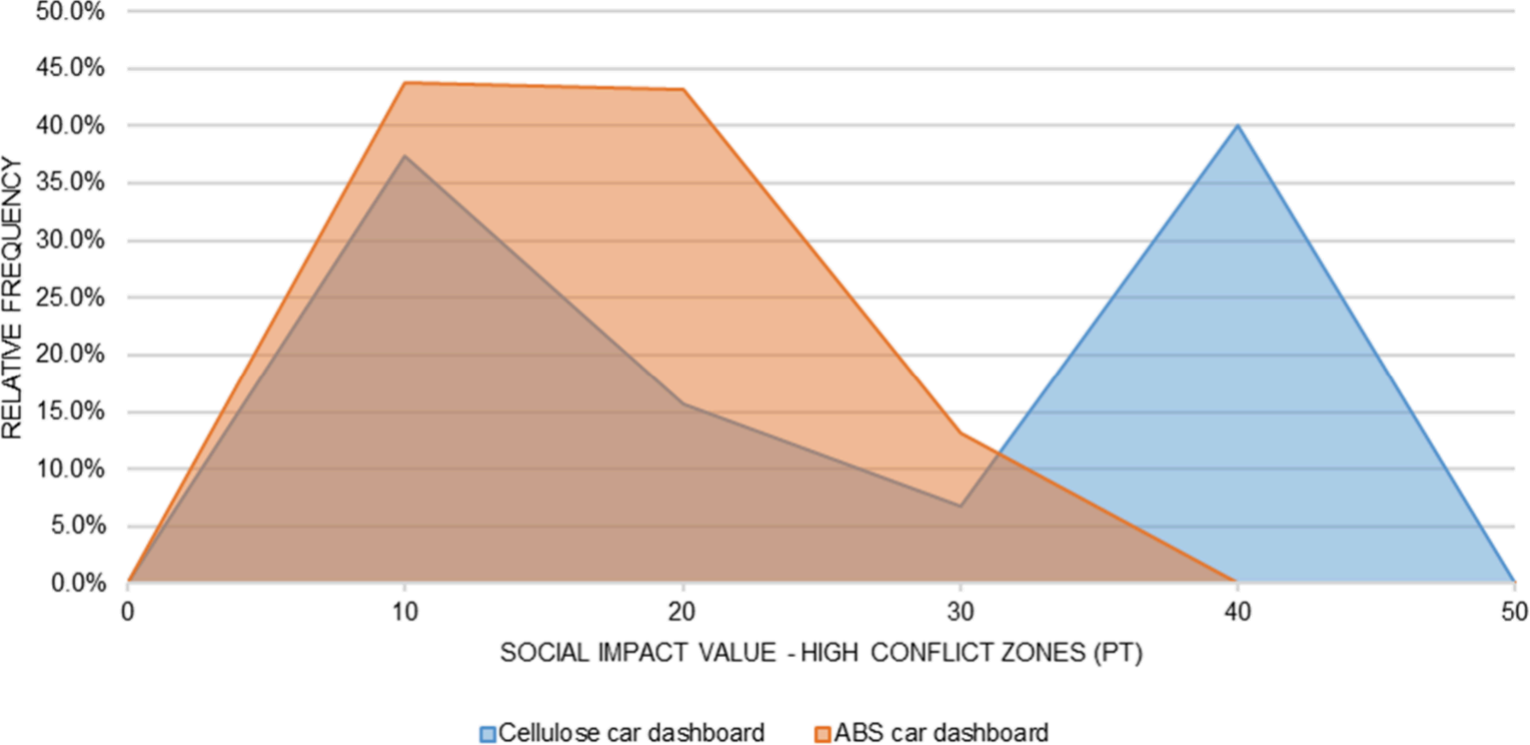
Probability distribution: High Conflict Zones (car dashboard).

Finally, in the High Conflict Zones subcategory (Figure 16), the cellulose distribution shows two distinct peaks at different impact levels (around 10 and 40 Pt). This pattern indicates that the simulated outcomes combine contributions from regions with different conflict risk levels in the SHDB. When uncertainty is applied to the CFs, part of the simulated values aligns with regions associated with lower conflict intensity, while the other part reflects regions classified with higher conflict risks. Although there is overlap with the ABS curve at lower values, most outcomes for the cellulose system fall in higher ranges, and the curve extends further to the right, resulting in a higher mean value. Welch’s *t* test confirmed a statistically significant difference between the materials, with a 95% confidence interval ranging from −8.46 to −7.70 Pt. Because zero is not included in this interval, the result supports a clear and consistent difference in social impact levels between the two options.

Figures 17 to 21 present the distributions of simulated values for the five social impact subcategories analyzed in the ship counter bar. Unlike what was observed for the car dashboard, in this system, the cellulose-based alternative showed systematically lower social impact values across all subcategories, indicating superior performance compared with the conventional gypsum material.

**Figure 17:**
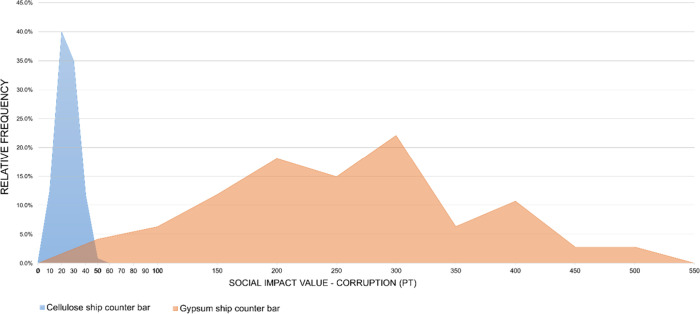
Probability distribution: Corruption (ship counter bar).

Although the difference between the two materials is very large, the stochastic analysis remains relevant because it confirms that this gap is not an artifact of the deterministic modeling. In cases where the performance difference is wide, the role of uncertainty analysis is to verify that the conclusion is insensitive to the variation introduced in the characterization factors. The results show that the hierarchy between the materials remains unchanged across all simulations, indicating that the difference is structurally robust and is not dependent on parameter uncertainty.

In the Corruption subcategory (Figure 17), the curve for the cellulose-based alternative is sharply concentrated in very low impact ranges, whereas the curve for the conventional gypsum system is broad, irregular, and spread across considerably higher values. The clear separation between the distributions illustrates the extent of the difference and is consistent with the statistical results. This result is supported by Welch’s *t* test, which confirmed a statistically significant gap between the two materials. The 95% confidence interval for the difference in means, from 208.74 to 213.95 Pt, does not include zero, reinforcing the conclusion that the cellulose-based material consistently performs better in this impact category.

In Occupational Toxics and Hazards (Figure 18), a similar pattern is observed: the cellulose-based alternative presents a sharply concentrated distribution at low impact values with minimal dispersion. In contrast, the curve for the conventional gypsum system is wider and clearly shifted toward higher impact levels. This visible divergence is statistically supported by Welch’s *t* test, with a 95% confidence interval for the difference in means ranging from 191.53 to 195.34 Pt. Since this interval lies entirely above zero, the result confirms a consistent and statistically significant advantage for the cellulose-based material.

**Figure 18:**
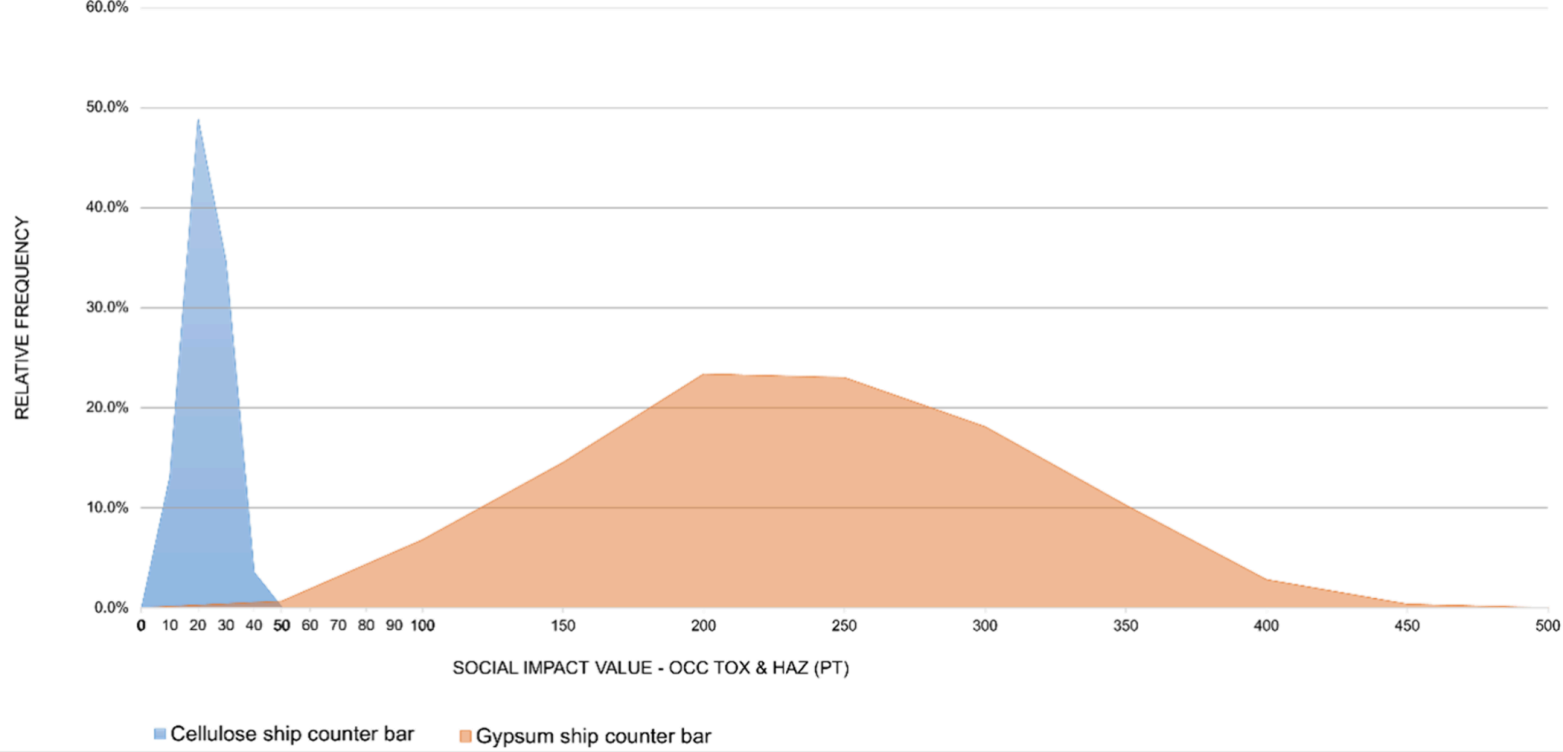
Probability distribution: Occupational Toxics and Hazards (ship counter bar).

In the Legal System subcategory (Figure 19), the distribution for the cellulose-based alternative is narrow and concentrated at low impact values, while the curve for the conventional gypsum system is broader and exhibits multiple peaks, indicating greater variability and generally higher social impact values. The statistical results support this observation: Welch’s *t* test confirmed a significant difference at the 95% confidence level, with a confidence interval for the difference in means ranging from 148.59 to 153.27 Pt. Because this interval does not include zero, it provides robust evidence that the cellulose-based option outperforms the conventional alternative in this category.

**Figure 19:**
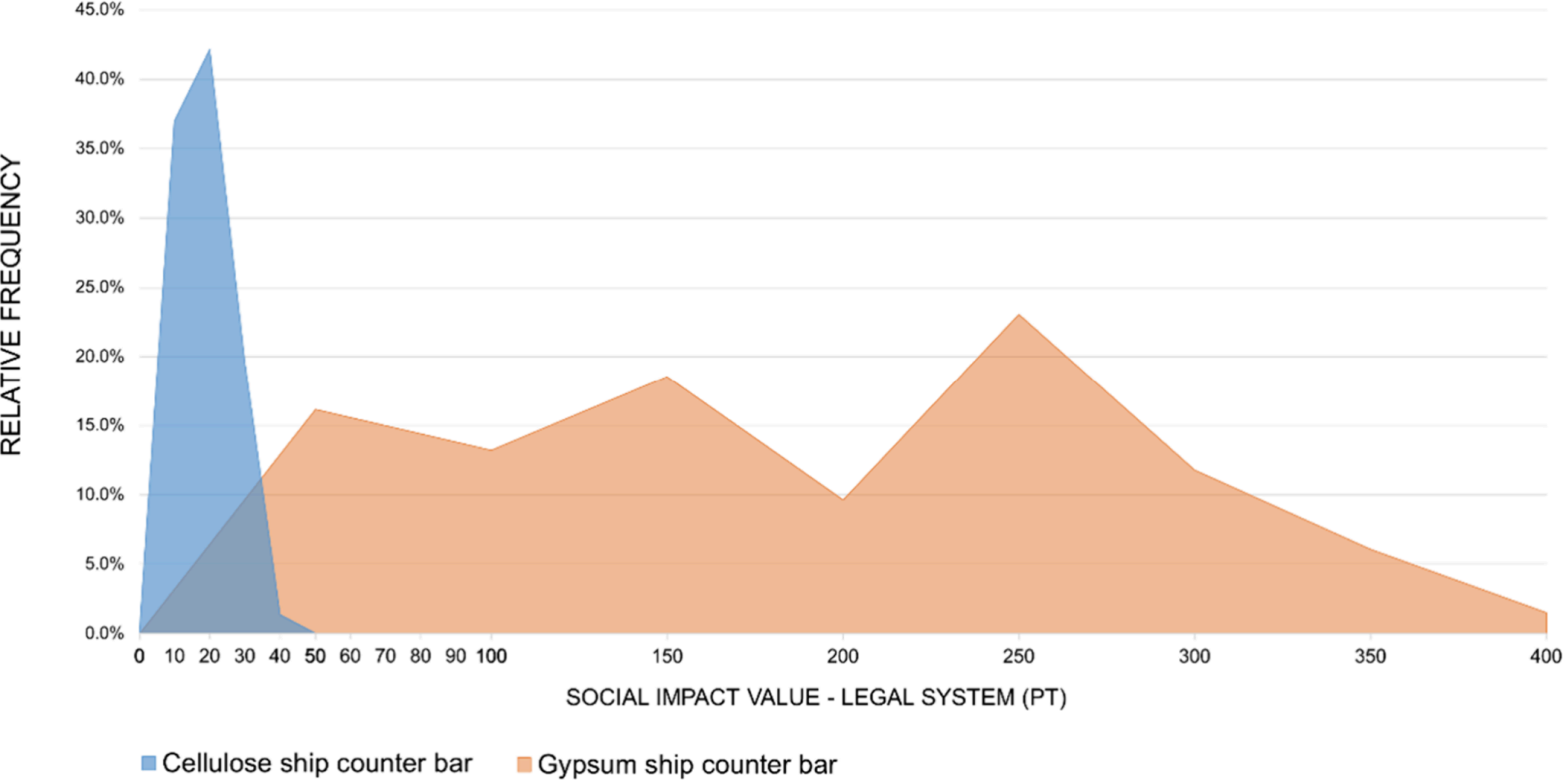
Probability distribution: Legal System (ship counter bar).

In the Injuries and Fatalities subcategory (Figure 20), a similar trend of lower social impact is evident for the cellulose-based alternative, which presents a sharply peaked distribution in the low-impact range. In contrast, the conventional gypsum material displays a broader distribution shifted to higher values with minimal overlap between the two curves. This pattern is statistically supported by Welch’s *t* test, which identified a significant difference at the 95% confidence level. The confidence interval for the difference in means ranges from 140.03 to 143.35 Pt and does not include zero, confirming that the cellulose-based material consistently outperforms the conventional option in this subcategory.

**Figure 20:**
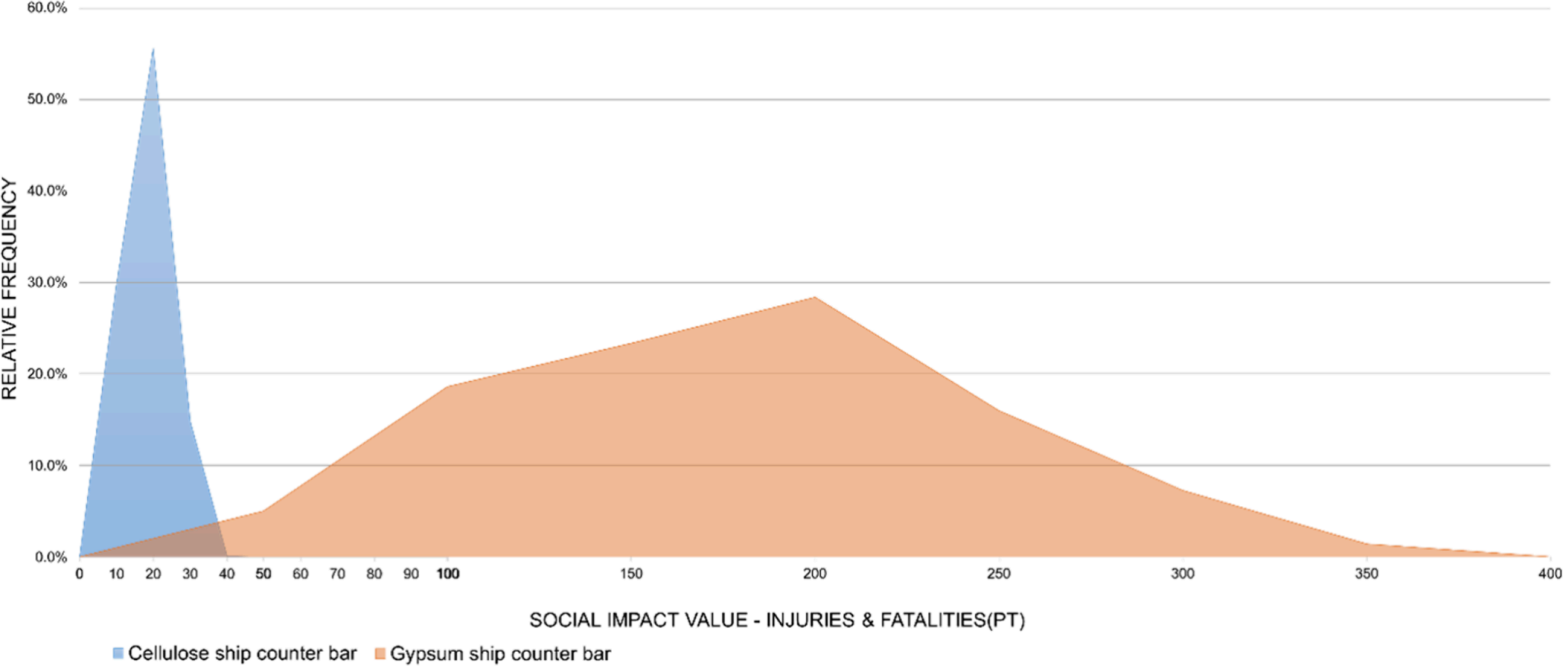
Probability distribution: Injuries and Fatalities (ship counter bar).

Finally, in the High Conflict Zones subcategory (Figure 21), the separation between the distributions is once again clear. The cellulose-based alternative exhibits a concentrated distribution at low impact values, while the conventional gypsum material presents a broader and more irregular curve, extending toward higher impact values. The statistical analysis using Welch’s *t* test confirmed that this difference is significant at the 95% confidence level. The confidence interval for the difference in means ranges from 219.35 to 227.59 Pt and does not include zero, indicating a consistent and significant advantage of the cellulose-based option in this social subcategory.

**Figure 21:**
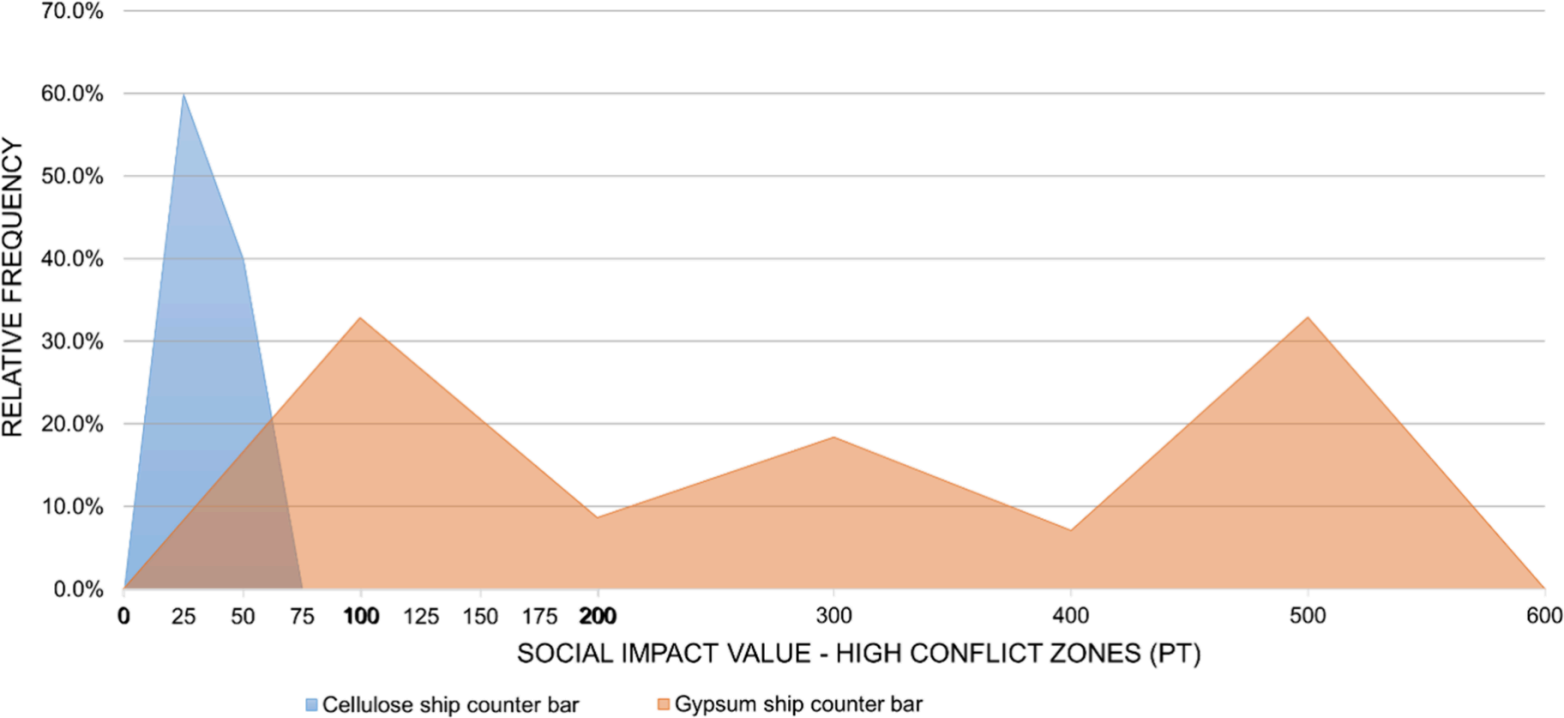
Probability distribution: High Conflict Zones (ship counter bar).

These distributions visually confirm the statistical results, showing that for the car dashboard, the cellulose-based alternative performed consistently worse in the most relevant subcategories. In contrast, for the ship counter bar, the cellulose-based alternative exhibits a consistently more favorable social impact profile than the conventional material across all of the analyzed subcategories.

## 4 Discussion

This study evaluated the influence of data uncertainty on the social performance comparison between cellulose-based materials and conventional materials. The results showed that the relative ranking of materials observed in the deterministic analysis was maintained when uncertainty was included through stochastic modeling. For the car dashboard, the cellulose-based alternative consistently presented higher social impact values across all analyzed subcategories, while for the ship counter bar, the cellulose-based material showed lower social impact values. The observation that these patterns remained stable under uncertainty indicates that the conclusions are statistically reliable, meaning that the differences are linked to structural characteristics of the supply chains rather than to uncertainty in the data inputs.

The opposite behavior of the cellulose-based material in the two product systems is explained by the structure of the dominant processes, sectors, and regions represented in each system rather than by the material itself. The cellulose supply chain remains essentially the same in both cases, so the differences arise from the ABS and gypsum systems. This system is dominated by chemical production in Italy, a sector region combination that shows comparatively lower risk levels in the most influential subcategories according to the SHDB. By contrast, the cellulose dashboard is dominated by pellet production in Finland, which concentrates most worker hours in sectors associated with medium to high risks for occupational toxics and hazards and occupational injuries. This distribution of labor and sector-level intensity explains why the total impacts of the cellulose dashboard are higher than those of the ABS.

In the ship counter bar, the much larger gap between the alternatives results from the processes that dominate the gypsum system. These processes involve very high volumes of worker hours in sectors such as polyurethane, polyester, and glass fiber production. In the SHDB, total impact is calculated by multiplying sector-level risk intensity by the number of worker hours, meaning that systems with large labor inputs in high intensity sectors generate much higher overall impacts. Although the cellulose-based system in the ship counter bar also includes sectors with elevated risk levels, the volume of worker hours is considerably smaller and concentrated in fewer high intensity sectors. This combination of sector intensity and labor distribution explains why the cellulose-based material performs worse than ABS in the dashboard but performs much better than gypsum in the ship counter bar. The divergence in results therefore reflects differences in the conventional comparison systems rather than inconsistencies in the social performance of the cellulose-based alternative.

The stochastic analysis did not aim to change the deterministic findings but to assess their stability under parameter uncertainty. This is a relevant methodological contribution because the differences in social impact between alternatives could theoretically diminish or reverse when accounting for uncertainty in the characterization factors. The fact that the ranking remained the same, with confidence intervals that did not include zero, indicates that the performance differences are stable rather than artifacts of deterministic modeling. At the subcategory level, the stochastic results reveal how uncertainty is distributed within each dimension of social performance, indicating which social issues are more sensitive to data uncertainty and where supply chain conditions exert the strongest influence.

Beyond comparing mean values, the stochastic results provide additional information that can be used directly to support decision-making. The quartiles show how impact values are distributed and make it possible to identify the dispersion associated with each alternative. This helps determine whether a small difference between materials is consistent or depends on high variability. When the interquartile range is narrow, the decision maker can see that the alternative shows predictable behavior even in the presence of uncertainty; when it is wide, the stability of the conclusion decreases, and the choice requires caution.

The graphs that show the distribution of simulated values allow a visual assessment of the robustness of the results. The absence of overlap between the distributions indicates that the difference between alternatives is maintained even when the characterization factors vary within defined uncertainties. When partial overlap exists, the decision maker can identify the degree of risk of preference reversal and assess whether that probability is acceptable for the application context.

The statistical tests used complement these elements. The Levene test identifies whether the variances between alternatives are comparable, while the Welch test verifies whether the means differ in a statistically significant way. This verification quantifies the reliability of the conclusion and provides a formal criterion for justifying the choice of an alternative to procurement teams, internal auditors, certification bodies, or design processes.

These results make it possible to translate the social comparison to operational information. Quartiles can be used to define safety margins; the assessment of overlap indicates the risk of preference reversal; and the statistical tests quantify the confidence associated with each choice. In this way, the stochastic analysis not only confirms the stability of the deterministic conclusions but also provides indicators that can be directly integrated into material selection processes, supplier evaluation, and the definition of social traceability criteria. The study also highlights methodological limitations. The results depend on the SHDB database, which aggregates social information from heterogeneous sources with varying levels of granularity. The characterization factors provided by the SHI method do not have documented derivation procedures, which introduces structural uncertainty.

Although these characterization factors are widely used in the SHI method, their undocumented origin means that the multipliers between risk levels may not reflect empirical or theoretical evidence. This implies that the absolute magnitude of the social impact values can vary depending on how these factors are defined. However, the stochastic modeling applied in this study makes it possible to evaluate whether this structural uncertainty affects the relative comparison between materials. By allowing the characterization factors to vary within the uncertainty ranges defined for each risk level, the simulations test whether different plausible realizations of these factors would change the ranking of the alternatives. In both case studies, the relative performance of the materials remained stable across all subcategories, indicating that the conclusions do not depend on the specific fixed multipliers used in the SHI method. In this sense, the uncertainty analysis does not eliminate the structural limitation but reveals that the comparative results are robust to variations in the characterization factors.

The uncertainty scoring of indicators was performed by a single evaluator following PEF data quality criteria adapted to the social context and even validated by two evaluators afterward introduces a degree of subjectivity. Besides that, the normalization factors and the equal weighting scheme used in the SHI method are also fixed parameters without documented derivation, and their use may introduce additional uncertainty that was not assessed in the present study. These limitations do not invalidate the results, but they indicate the need for future work to refine uncertainty modeling, explore sensitivity analysis of scoring decisions, and examine the influence of the SHI normalization factors and weighting scheme, as well as incorporate stakeholder-based validation.

Despite these limitations, the integration of uncertainty modeling into S-LCA provides a more transparent basis for material selection. By showing not only the expected social impact levels but also the variability associated with them, the approach supports early stage decision-making with an explicit consideration of reliability. The methodology can be extended to other product systems and sectors, including contexts where data availability is limited, contributing to more grounded assessments of social performance in biobased material innovation.

The approach also has practical implications. For companies comparing materials, the method allows not only the calculation of social impact values but also the evaluation of how stable these values remain when data uncertainty is considered. This helps teams decide whether a difference in social performance between two materials is consistent or depends on uncertain inputs. In practice, the results can support supplier selection, procurement strategies, and the early stage screening of alternative materials in design and development. For policymakers and certification bodies, the approach highlights which parts of the supply chain are more exposed to labor conditions, governance, or occupational risks and where regulatory or monitoring efforts could have a measurable effect. For research and innovation in biobased materials, the results show that social performance depends on the specific supply chain context rather than on the material category itself. This means that new materials should not be assumed to have better or worse social outcomes by default. Instead, their performance needs to be assessed, considering where and how they are produced, how labor is organized, and the governance conditions of the regions involved.

## 5 Conclusions

This study proposes a methodology for uncertainty analysis in S-LCA, showing that integrating uncertainty modeling into S-LCA can enhance the robustness, credibility, and interpretability of social performance evaluations. By combining deterministic and stochastic approaches, the proposed methodology allows for a more comprehensive understanding of how uncertainty affects comparisons between alternative materials. The application to two case studies, an automotive dashboard and a ship counter bar, showed that even under data uncertainty, the cellulose-based alternatives exhibited consistent performance patterns, either favorable or unfavorable depending on the context. Statistical analyses, including Levene’s and Welch’s *t* tests, confirmed the significance of the observed differences at the 95% confidence level, reinforcing the reliability of results despite limitations in data availability or quality.

Moreover, the proposed methodology proved to be practical and adaptable, requiring only accessible tools, such as Excel and @Risk, to be applied. It can be applied to early stage development processes, supporting more socially responsible material choices, to products and process comparison, and to retrofit value chains. The methodology also enhances the decision-making capacity of multiple stakeholder groups by providing both impact results and a quantifiable measure of the result reliability. The application of the methodology to two distinct product systems in different industrial sectors, automotive and maritime, demonstrates its flexibility and potential for use across diverse industrial contexts. These results suggest that the approach can be adapted to diverse value chains; however, applying it to a broader range of sectors and materials would be valuable to confirm its robustness and general applicability.

Future work should further refine this approach by incorporating other sources of uncertainty, such as model assumptions and context-specific variations, as well as by combining it with stakeholder engagement and qualitative insights to improve the relevance and depth of S-LCA applications in real-world contexts.

In addition, future research could explore differentiated weighting schemes for data quality assessment, for example, through multicriteria decision analysis (MCDA) or expert-based weighting approaches, to complement the PEF-based evaluation and further improve the sensitivity of the uncertainty scoring system.

## Supplementary Material



## Notes

Primary data used to model the product systems in SimaPro, detailed tables presenting the social indicators and their uncertainty scores, and the data source quality assessment (PDF)
