# Supplementary material for: Advancing Social Life Cycle Assessment: A Novel Approach to Uncertainty Analysis
Source: Ind Eng Chem Res. 2026 Jan 26;65(5):2813–35. doi: 10.1021/acs.iecr.5c03058 (PMC12903759; doi:10.1021/acs.iecr.5c03058)
Supplement: Supplementary file 1 [file ie5c03058_si_001.pdf]

Supporting Information for Publication

Title: Advancing Social Life Cycle Assessment: A Novel Approach to Uncertainty Analysis

Beatriz Cassuriaga[a], Andreia Santos[a], Ana Carvalho[a]*

[a] CEGIST – Centro de Estudos de Gestão do Instituto Superior Técnico, Universidade de Lisboa, Av. Rovisco Pais, 1049-101 Lisboa, Portugal
*Email: anacarvalho@tecnico.ulisboa.pt

Appendix S.1 – Primary data collected

Table S1. Primary data collected for the cellulose-based car dashboard manufacturing stage.

Material	Sector	% Qty	% Cost	Country
**Cellulose-based Car Dashboard**				
Cellulose-based material Pellets	chemical products	39.02%	95.92%	Finland
Water	water	0.43%	0.24%	Italy
Kaolin	manufacture non-metallic mineral products	0.13%	0.00%	Italy
Lime	manufacture non-metallic mineral products	0.15%	0.01%	Italy
Lubricating oil	chemical products	0.12%	0.29%	Italy
Malusil	chemical products	0.02%	0.02%	Italy
Polyethylene	chemical products	0.07%	0.02%	Italy
Polypropylene	chemical products	0.14%	0.07%	Italy
Solvent, organic	chemical products	1.75%	1.19%	Italy
Titanium dioxide	manufacture non-metallic mineral products	0.08%	0.03%	Italy
Electricity	electricity	58.1%	2.21%	Italy

Table S2.Primary data collected for the cellulose-based car dashboard end of life stage.

Material	Sector	% Qty	% Cost	Country
**Cellulose-based Car Dashboard**				
Landfill	water	41.00%	49.8%	Italy
Incineration	water	59.00%	50.2%	Italy

Table S3. Primary data collected for the gypsum ship counter bar manufacturing stage.

Material	Sector	% Qty	% Cost	Country
**Gypsum Ship Counter Bar**				
**GRG (Glass Reinforced Gypsum)**				
Gypsum	manufacture non-metallic mineral products	11.73%	0.28%	Finland
Glass fiber	chemical products	0.65%	0.16%	Finland
Epoxy resin, liquid (to coat the mold)	chemical products	0.25%	0.48%	Finland
Polyurethane, rigid foam	chemical products	35.62%	22.78%	Finland
Tap water	water	3.53%	0.06%	Finland
**GRP (Glass Reinforced Plastic)**				
Polyester	chemical products	8.03%	4.5%	Finland
Glass fiber	chemical products	4.32%	1.3%	Finland
Epoxy resin, liquid (to coat the mold)	chemical products	0.25%	0.48%	Finland
Polyurethane, rigid foam	chemical products	35.62%	22.78%	Finland

Table S4. Primary data collected for the gypsum ship counter bar end of life stage.

Material	Sector	% Qty	% Cost	Country
**Gypsum Ship Counter Bar**				
Landfill	water	95.00%	91.28%	Finland
Recycling	water	5.00%	8.72%	Finland

Table S5. Primary data collected for the cellulose-based ship counter bar manufacturing stage.

Material	Sector	% Qty	% Cost	Country
**Cellulose-based Ship Counter Bar**				
**Material drying and handling**				
Cellulose-based material Pellets	chemical products	12.27%	90.03%	Finland
Electricity (Heating the granulate)	electricity	1.23%	0.14%	Finland
**3D printing process**				
Electricity (Pellet melting)	electricity	9.82%	1.12%	Finland
Electricity (Process control)	electricity	6.13%	0.7%	Finland
Electricity (Bed Heating)	electricity	18.4%	2.09%	Finland
Electricity (Motion)	electricity	30.67%	3.48%	Finland
**Prototype line**				
Electricity (Finishing)	electricity	21.47%	2.44%	Finland

Table S6. Primary data collected for the cellulose-based ship counter bar end of life stage.

Material	Sector	% Qty	% Cost	Country
**Cellulose-based Ship Counter Bar**				
Landfill	water	41.00%	49.8%	Finland
Incineration	water	59.00%	50.2%	Finland

Appendix S2 – Example of Characterization, Normalization, and Weighting in the Deterministic Impact Assessment

Table S7. Example of characterization, normalization, and weighting calculations for the Corruption subcategory.

**System**	Cellulose Car Dashboard					
**Subcategory**	Corruption					
**Indicator**	Overall Corruption					
**Risk Level**	LCI	Caracterization Factor (CF)	LCI x CF	Characterized Value (CV)	Normalization Factor (NF)	Normalized and Weighted Value (CV x NF)
**LR**	0,098	0,4	0,039	16,932	10,0	169,32
**MR**	0,414	4	1,655			
**HR**	0,210	20	4,197			
**VH**	0,276	40	11,041			

This table illustrates the implementation in Excel of the characterization, normalization, and weighting steps for the Corruption subcategory of the cellulose car dashboard system. The life cycle inventory (LCI) values were exported from SimaPro after modeling the system using the Social Hotspot Database (SHDB). Specifically, the social flows corresponding to worker hours were quantified based on the cost data of materials, utilities, and processes, which were assigned to the corresponding economic sectors and regions as described in Step 2. Using the SHI method implemented in SimaPro, the total worker hours associated with the Corruption indicator were obtained and disaggregated by risk level (low, medium, high, and very high), according to the characterization factors provided by the SHDB. These disaggregated worker-hour values constitute the LCI input used for the characterization calculations in Excel. Characterized results were obtained by multiplying the LCI values by the corresponding characterization factors for each risk level and summing the results. Normalization was performed by multiplying the characterized value by the normalization factor provided by the SHI method. As the SHI method assigns a weight equal to 1 to all subcategories, the normalized and weighted value is the same.

Appendix S.3 – Data Source Quality Assessment

Table S8. Criteria adapted from the Product Environmental Footprint (PEF) method for assessing data quality in S-LCA

Criterion (adapted from PEF)	Description in the S-LCA context	Evaluation guidance (examples of scores 1, 3, 5)
**Completeness**	Assesses how well the source covers the relevant aspects of the social indicator being analyzed (e.g., coverage by country, sector, gender, age, etc.).	1 – Broad, detailed data with multiple relevant dimensions (e.g., disaggregated by sex and sector).<br>3 – Moderately complete data, with relevant gaps (e.g., missing sector or population group).<br>5 – Partial, generic, or poorly scoped data.
**Methodological appropriateness & consistency**	Evaluates whether the data collection and processing methods are clear, institutionally recognized, systematic, and consistently applied.	1 – Well-documented methodology, statistically sound and institutionally recognized.<br>3 – Partially clear or applied with limitations.<br>5 – Opaque methodology, self-reported data without technical control or documentation.
**Technological representativeness**	Reflects how well the data represent the technical, occupational, or sectoral context of the indicator.	1 – Specific data by occupation, sector, or function (e.g., agricultural workers, manufacturing).<br>3 – Generic data by broad economic activity.<br>5 – National data with no sectoral distinction or highly aggregated.
**Geographical representativeness**	Assesses whether the data refer to the country or local context relevant to the analysis.	1 – Country-specific or regionally disaggregated data.<br>3 – Regional or global data with adjustments or limitations.<br>5 – External or generic data without adaptation to local context.
**Time-related representativeness**	Assesses whether the data are recent and aligned with the period of analysis, and whether they are regularly updated.	1 – Recent data with regular updates (within the last 5 years).<br>3 – Data older than 8–10 years or with unclear updates.<br>5 – Very outdated data or unknown collection date.
**Precision**	Evaluates the confidence level in the data source based on methodological rigor, sample representativeness, and transparency.	1 – High precision based on probabilistic sampling, quality control, and external validation.<br>3 – Uncertain precision, limited sample or no margin of error stated.<br>5 – Low precision or presumed high uncertainty (e.g., estimates or unverifiable sources).

Note: *Scores range from 1 (excellent) to 5 (very poor), as defined in the Product Environmental Footprint (PEF) method (Manfredi et al., 2012).*

Table S9. Data Source Quality Assessment

Subcategory	Indicator	Description	Full Reference	Completeness	Methodological appropriateness & consistency	Technological representativeness	Geographical representativeness	Time-related representativeness	Precision
**1A Wage**	Risk that Avg Wage is Below Country Minimum Wage	The average salary earned by workers in a specific country and sector, used to calculate the number of worker hours associated with production.	United Nations Industrial Development Organization (UNIDO). 2018. Retrieved from: http://www.unido.org	2	1	2	1	1	2
				Covers key economic indicators relevant for average wage calculation, though lacks occupational detail.	Methodology based on ISIC classification; highly consistent with international standards.	Good sectoral representativeness, though lacks occupational or process-level detail.	Broad national coverage by country; official, comparable data.	Updated annually; 2018 data is recent.	Good statistical precision; aggregated by national institutions.
			ILOSTAT. 2018. ILOSTAT Internet. Retrieved from: https://www.ilo.org/ilostat/	1	1	2	1	1	1
				Broadly covers average monthly and hourly wages, disaggregated by sex, sector, and occupation.	Strong and harmonized statistical methodology by the ILO, widely recognized.	Good representation of sectors and occupations, though not process-specific.	Wide global coverage with consistent national data.	Regularly updated; 2018 edition reflects the relevant period.	High statistical precision, based on official records and representative surveys.
			Paylab. 2018. Retrieved from: https://www.paylab.com/OM/salaryinfo	3	4	4	4	3	4
				Data refers only to Oman and is limited to wage reporting, lacking other relevant social or occupational dimensions.	Methodology lacks transparency; relies on user self-reporting and does not follow recognized statistical standards.	Technological representativeness is weak, with no distinctions between different activities or technologies.	Despite national coverage being claimed, there is no sampling control or evidence of population-level representativeness.	Data is from 2018, but no information is available about update frequency or continuity.	Precision is weak due to absence of probabilistic sampling, external validation, or uncertainty estimates.
			OECD. 2018. Employee compensation by activity (indicator). Retrieved from: https://data.oecd.org/earnwage/	2	1	2	2	1	1
				Provides detailed data on employee compensation by economic activity, but lacks broader social context.	Robust and standardized OECD methodology, with clear documentation aligned with international statistical standards.	Good sectoral representativeness based on ISIC, though lacking detailed occupational breakdown.	Broad coverage for OECD countries, but limited data availability for non-member countries.	Data is regularly updated; the 2018 edition reflects a recent period.	High statistical precision, based on validated national sources compiled by OECD.
				3	4	4	3	3	4

		Jeune Afrique. 2018. Retrieved from: https://www.jeuneafrique.com/emploi-formation/609859/salaires-au-maroc-le-secteur-public-paie-mieux-que-le-prive/	Presents average wages for public vs. private sectors in Morocco, but lacks disaggregation by sector, occupation, or wage brackets.	Methodology is not described; no reference to primary data sources or collection procedures.	Limited technological representativeness; no differentiation by occupation or production activity.	Focused on Morocco, but no evidence of representative sampling at the national level.	Data refers to 2018, but no information on update frequency or temporal resolution.	Weak precision; figures are generic and lack statistical detail or error margins.
			3	4	4	3	4	4
		Afghanistan profile. Retrieved from: http://www.bamf.de/SharedDocs/MILo-DB/EN/Rueckkehrfoerderung/Laenderinformationen/Informationsblaetter/cfs_afghanistan-dl_en.pdf	Includes general references to average wage levels, but lacks sectoral, occupational, or methodological detail.	No explanation of the data collection process; the document is a migration-focused report, not a statistical dataset.	Limited representativeness; no differentiation by economic sector, job type, or technology.	National focus on Afghanistan, but no indication of sampling or statistical coverage.	Data is from 2014 and outdated for current assessments.	Low precision: no clear source, no margin of error, and no data quality documentation.
			1	1	2	1	1	1
		Eurostat. 2018. Retrieved from: https://ec.europa.eu/eurostat/web/microdata/european-union-labour-force-survey	Covers wages, working conditions, hours worked, occupation, sector, and other essential social variables.	Rigorous statistical methodology standardized by Eurostat and validated by national institutes.	Good representativeness by sector and occupation, though not explicitly differentiated by production technology.	Full coverage of EU countries with comparable and representative data based on sampling.	Continuously collected and regularly updated data; 2018 edition accurately reflects the reference period.	High statistical precision with probabilistic sampling, cross-validation, and transparent documentation.
			1	1	2	1	1	1
		INSEE. 2018. Retrieved from: https://www.insee.fr/en/accueil	Provides detailed coverage of wages by sector, occupation, qualification, age, and gender, including comparisons to the minimum wage.	Robust methodology using administrative records and official surveys, following French and EU statistical standards.	High representativeness by occupation and sector, though not broken down by production technology.	Full national coverage with representativeness ensured through sampling and administrative sources.	Regularly updated data; 2018 edition accurately represents the reference period.	High statistical precision with validated official data, controlled sampling, and known error margins.
			2	2	3	2	3	3
Risk that Sector Avg Wage is below Living Wage	An estimated wage required for a worker to afford basic living costs, including food, housing, transport, health, education, and taxes.	WageIndicator.org; wages in context. Retrieved from: https://wageindicator.org/salary/wages-in-context	Provides living wages and average wages by sector for several countries, but with limited sectoral and country coverage in lower-income regions.	Methodology is moderately well documented, based on local costs of living, but does not always follow official standards.	Technological representativeness is average; some sector differentiation but no process-level detail.	Reasonable geographic coverage, with stronger representation in OECD countries.	Some data is regularly updated, but others are outdated or lack a clear reference year.	Precision varies by country; based on local estimates with limited transparency on uncertainty or error margins.
			3	3	3	3	3	3
Risk that Sector Avg Wage is	A wage value adjusted to the economic development level of each country, used as	Sweatfree Contracting Ordinance (Administrative Code). Retrieved from:	Methodology provides a	Derived from public procurement policy,	Limited representativeness;	Applies country-level adjustments,	Data is from 2018 with no evidence of	Precision is moderate to low; it

	below Sweatered Wage	a conservative benchmark for fair compensation.	https://sfgov.org/olse/sweatfree-contracting-ordinance	benchmark "sweatfree wage" based on adjusted purchasing power, but original data is US-based and extrapolated globally.	adapted internationally; limited transparency on modeling or equations used.	considers national income levels but does not differentiate technologies, sectors, or occupations clearly.	but lacks direct empirical data from most countries; coverage is modeled.	continuous updates; adjustments are static and based on broad economic indicators.	is a benchmark, not a measured dataset, and lacks uncertainty estimates or cross-validation.
**1B Poverty**	Percent of population living under the relevant poverty line	The percentage of the population living below the international poverty lines of $1.90, $3.20, or $5.50 per day, depending on the country's income classification, used to assess the risk of poverty in different countries.	World Bank. 2017. *World Development Indicators*. Retrieved from: http://data.worldbank.org/indicator / and https://data.worldbank.org/topic/poverty	1<br>Fully covers the expected data flows for poverty assessment across all income levels.	1<br>Consistent methodology based on standardized household microdata and PPP-adjusted thresholds.	2<br>Technology is not explicitly considered, but poverty is linked to sectoral and structural economic conditions.	1<br>Global coverage with internationally comparable data.	1<br>Regular updates; the 2017 edition reflects recent data for most countries.	1<br>High statistical precision, based on nationally validated household survey data.
**1D Child Labor**	Risk of child labor by sector (qualitative)	The identification of sectors in each country where child labor is reported, based on qualitative assessments, used to classify the sectoral risk level.	U.S. Department of Labor. *Findings on the Worst Forms of Child Labor* and *List of Goods Produced by Child Labor or Forced Labor*. Retrieved from: https://www.dol.gov/agencies/ilab/resources/reports/child-labor	2<br>The source covers many countries and sectors with records of child labor, focusing on specific products and supply chains.	3<br>The methodology is qualitative and aligned with the goal, but lacks statistical standardization and replicable criteria, reducing consistency across countries.	3<br>Sectors are identified, but no breakdown by technology or production process is provided.	2<br>Broad international coverage focused on developing countries, though depth varies with data availability.	2<br>Data is current up to 2017, but no clear update frequency by country or sector.	3<br>Precision is limited due to lack of quantitative data, error margins, or statistical validation. Information is qualitative and varies in reliability.
			International Trade Union Confederation. 2012. Retrieved from: http://www.ituc-csi.org/documents.html	4<br>The source mentions sectors and countries with child labor, but coverage is irregular, unsystematic, and should be used cautiously.	3<br>Methodology is implicit, based on union reports and denunciations, with no clear criteria or consistent application.	4<br>Weak technological representativeness; sectors are referenced broadly, without detailing processes or occupations.	3<br>International scope, but uneven — countries with stronger union presence are more documented.	4<br>Data is from 2012 with no sign of updates or continuity, which limits temporal relevance.	4<br>Low precision: no statistical validation, error margins, or structured sampling; useful for alert purposes only.
**1E Forced Labor**	Overall Forced Labor in Country	The estimated percentage of a country's population subjected to modern slavery or forced labor, used to assess the national risk level of forced labor.	Global Slavery Index. 2016. Retrieved from: http://www.globalslaveryindex.org /	2<br>The source covers most countries and provides national estimates of forced labor, though without sectoral disaggregation.	2<br>The methodology is well-structured, combining primary survey data and econometric models, and is	3<br>The data is aggregated at the national level, lacking distinction by productive sector or technology.	2<br>Global coverage with varying levels of confidence depending on the availability and quality of national data.	2<br>The source is periodically updated (2016, 2018, 2023), even if SHDB uses the 2016 edition.	2<br>Precision is relatively good, with transparent methods and representative data, though statistical error

					consistent with the indicator's purpose.				margins are not explicitly reported.
**1F Excessive WkTime**	Percent of Population working >X hrs. per week, >60 hrs. per week	The percentage of the workforce working more than 60 hours per week, used to assess the risk of excessive working time in a country.	International Labor Organization, ILOSTAT. 2014. Retrieved from: http://www.ilo.org/ilostat	1	1	2	1	2	1
				The database extensively covers working hours with valuable disaggregation and national coverage.	Well-established methodology, widely applied and harmonized by the ILO.	Good representation by sector and occupation, though lacking technological granularity.	Strong global coverage with statistical standardization across countries.	Data from 2014; useful but potentially outdated in some countries.	High statistical precision, based on official records and representative surveys.
			International Labor Organization. 2014. Key Indicators of the Labor Market (KILM). Retrieved from: http://www.ilo.org/kilm	1	1	2	1	2	1
				The dataset broadly covers population working >60 hrs./week, with good national disaggregation.	Clear and consolidated methodology, with harmonized application across countries.	Good sectoral representation, though not differentiated by applied technology.	Global coverage with strong comparability across countries.	Data from 2014, though frequently updated and part of time series at the time.	High precision based on official national statistics harmonized by the ILO.
**1G Freedom of Assoc**	Overall risk of Freedom of Association	The assessment of the risk that workers in a country face limitations on their rights to form unions, bargain collectively, and strike, based on violations reported by international labor organizations.	International Trade Union Confederation. 2018. *The Global Rights Report 2018: ITUC Global Rights Index – The World's Worst Countries for Workers*. Retrieved from: https://www.ituc-csi.org/ituc-global-rights-index-2018	3	3	4	2	2	4
				General country-level coverage, lacking full institutional detail.	Based on complaints and qualitative assessment, with potential bias.	No differentiation by sectors or production processes.	Good country coverage, but depth varies.	Annual publication, recently updated.	High subjectivity; no statistical error metrics.
			International Labour Organization. 2018. ILOSTAT Database – Industrial Relations. Retrieved from: https://www.ilo.org/ilostat	2	2	3	2	2	2
				Broad legal data, with gaps in some countries.	Methodology based on national law and international conventions.	Country-level data, no technological breakdown.	Good global coverage, with differences in availability.	Periodically updated, though uneven among countries.	Consistent, based on official sources, without statistical uncertainty.
**1H Migrant Labor**	Evidence of Risk to Migrant Workers - Qualitative	The qualitative assessment of risks faced by migrant workers in a country, including exposure to exploitation, discrimination, unsafe working conditions, and lack of access to legal protections, based on reports from international organizations.	U.S. Department of State. 2017. *Country Reports on Human Rights Practices for 2017*. Retrieved from: https://www.state.gov/reports/2017-country-reports-on-human-rights-practices/	3	3	4	2	2	4
				Covers migration issues, but lacks specific focus on migrant workers or sectoral details.	Based on diplomatic reports and observations, lacking systematic methodology.	National-level information, no sectoral or occupational granularity.	Broad coverage, but uneven depth and possible political bias.	Annual updates; 2017 data reflects context of the time.	Qualitative and descriptive, no quantitative metrics or statistical precision.
			Organization for Economic Co-operation and Development (OECD) and Migrant Integration Policy Index (MIPEX). 2015. Indicators of immigrant integration. Retrieved from: http://www.mipex.eu/	2	2	4	2	3	3
				Broad indicators on migration policy and integration.	Structured methodology with clear criteria.	No occupational or technological breakdown.	Good coverage of OECD and partner countries.	2015 data, limited recent updates.	Qualitative structure without formal statistical precision.

**1I Social Benefits**	Overall risk of inadequate social benefits	The risk that workers in a country may lack access to adequate social benefits such as healthcare, retirement, disability, dependents' benefits, survivors' benefits, wage insurance, paid sick leave, maternity leave, and parental leave.	World Legal Rights Data Centre, McGill Institute for Health and Social Policy, 2015. Data also supplemented by ILO NATLEX, World Bank's Doing Business Law Library, Lexadin World Law Guide, WorldLII databases, Social Security Programs Throughout the World (SSPTW), ILO Working Time Database, ILO Maternity Protection Database, and WABA's Status of Maternity Protection by Country.	2	2	4	2	3	3
				Covers multiple legal dimensions of social benefits.	Legal analysis with consistent criteria.	No occupational or sectoral breakdown.	Good country coverage with accessible legal data.	2015 data; some risk of outdated information.	Structured qualitative assessment without statistical effectiveness data.
**1J Labor Laws Convs**	Number of Labor Laws by Sector	The number of labor laws recorded in a country, including sector-specific regulations covering industries such as agriculture, mining, manufacturing, services, transportation, and domestic work.	International Labour Organization (ILO). 2010. *NATLEX Database of National Labour, Social Security and Related Human Rights Legislation*. Retrieved from: http://www.ilo.org/dyn/natlex/natlex_browse.byCountry?p_lang=en	2	2	3	2	4	3
				Broad legal coverage across sectors.	Structured by official legal documents with clear criteria.	Covers sectors but not technologies.	Broad national coverage with variability by country.	2010 data; potentially outdated.	Reliable legal source without practical enforcement metrics.
**1K Discrimination**	Prevalence of discrimination in the workplace (qualitative)	The qualitative assessment of the existence and frequency of discrimination in the workplace based on national constitutions, laws, and observed reports of discriminatory practices.	U.S. Department of State. 2017. *Country Reports on Human Rights Practices for 2016*. Retrieved from: http://www.state.gov/j/drl/rls/hrrpt/humanrightsreport/index.htm#wrapper	3	3	4	2	3	4
				Variable coverage by country, lacking structured framework.	Based on reports and official documents, not standardized.	No differentiation by productive sector or occupations.	Broad geographic scope with inconsistent depth.	2016 data; possibly outdated.	Qualitative assessment with no statistical basis.
**1L Unemployment**	Unemployment percentage at sector level	The percentage of unemployment in specific sectors within a country, used to assess sector-specific unemployment levels and identify areas of the labor market most affected by unemployment.	ILO Laborsta (2008). Tables 3A&3D: Unemployment, general level and by economic activity. Retrieved from: http://laborsta.ilo.org	2	1	3	2	3	2
				Good coverage of economic sectors.	Established statistical methodology by ILO.	Sector-level data, not process-specific.	Broad global coverage.	Data maintained and updated via ILOSTAT.	Based on official and representative data.
				3	1	4	3	5	3

**2A Occ Tox & Haz**	Disability-adjusted life years due to occupational-related Lung Cancer	The estimated number of healthy years lost due to work-related lung cancer, used to assess the burden of disease from exposure to occupational carcinogens.	Driscoll, T., Nelson, D. I., Steenland, K., Leigh, J., Concha-Barrientos, M., Fingerhut, M., & Prüss-Üstün, A. (2005). The Global Burden of Disease Due to Occupational Carcinogens. American Journal of Industrial Medicine, 48, 419–431. Retrieved from: http://www.cdc.gov/niosh/nas/rdrp/appendices/chapter5/a5-6.pdf	Includes global burden estimates for lung cancer but lacks disaggregation by country or sector.	Peer-reviewed epidemiological model using DALYs, consistent with WHO burden methodologies.	Does not specify occupational categories or types of industrial exposure in detail.	Global estimates with limited applicability to specific regional or national contexts.	Data from 2005 with no clear update or revision indicated since.	Based on modeled estimates with acknowledged uncertainty but from reputable source.
				3	1	4	3	5	3
	Overall Occupational Cancer Risk - loss of life (DALYs)	The total disability-adjusted life years lost due to all types of occupational cancers, providing a measure of mortality and morbidity related to hazardous workplace exposures.	Driscoll, T., Nelson, D. I., Steenland, K., Leigh, J., Concha-Barrientos, M., Fingerhut, M., & Prüss-Üstün, A. (2005). The Global Burden of Disease Due to Occupational Carcinogens. American Journal of Industrial Medicine, 48, 419–431. Retrieved from: http://www.cdc.gov/niosh/nas/rdrp/appendices/chapter5/a5-6.pdf	Includes global burden estimates for occupational cancers, but lacks sector or country-level disaggregation.	Robust peer-reviewed DALY-based methodology aligned with WHO burden of disease framework.	Cancer risks are not broken down by occupation or industrial process.	Global scope; region-specific variation not accounted for in detail.	Based on 2005 data with no clear indication of update.	Derived from epidemiological modeling with some uncertainty.
				3	2	3	3	5	3
	Overall Occupational Noise Exposure Risk	The estimated burden of disease associated with long-term exposure to occupational noise, used to assess the health impacts of hearing loss at work.	World Health Organization. (2004). Occupational noise: Assessing the burden of disease from work-related hearing impairment at national and local levels. Geneva, Switzerland: Concha-Barrientos, M., Campbell-Lendrum, D., & Steenland, K. Retrieved from: http://www.who.int/quantifying_ehimpacts/publications/en/ebd9.pdf	Covers global burden estimates for occupational hearing loss but not disaggregated by economic sector.	WHO-compliant DALY framework with consistent modeling.	Generalized treatment of noise exposure; lacks sector- or equipment-specific variation.	Global estimates without country-level specification.	Published in 2004 with no signs of regular updates.	Based on modeling with reference data but limited empirical resolution.
				2	2	1	2	2	2
**2B Injuries & Fatalities**	Fatal injuries by sector	The number of fatal occupational injuries reported by economic sector, used to estimate the sectoral risk of death due to work-related accidents.	International Labour Organization. Statistics and databases. Retrieved from: https://www.ilo.org/global/statistics-and-databases/lang--en/index.htm.	Provides broad coverage of fatal and non-fatal injuries by sector, though completeness may vary by country due to gaps in national reporting systems.	Based on standardized ILO definitions and collection frameworks, but national-level data may follow different reporting protocols, affecting global methodological consistency.	Covers occupational injuries and fatalities with sector-specific disaggregation across countries, ensuring strong alignment with real-world sectoral conditions.	Global dataset with national-level data coverage.	Data are regularly updated and reflect recent reporting cycles.	Based on official records and standardized data submissions from countries.
				2	2	1	2	2	2

	Non-Fatal Work Related injuries by sector	The incidence of non-fatal injuries occurring in the workplace, disaggregated by sector, to assess risks of physical harm and safety deficiencies.	International Labour Organization. Statistics and databases. Retrieved from: https://www.ilo.org/global/statistics-and-databases/lang--en/index.htm.	Provides broad coverage of fatal and non-fatal injuries by sector, though completeness may vary by country due to gaps in national reporting systems.	Based on standardized ILO definitions and collection frameworks, but national-level data may follow different reporting protocols, affecting global methodological consistency.	Covers occupational injuries and fatalities with sector-specific disaggregation across countries, ensuring strong alignment with real-world sectoral conditions.	Global dataset with national-level data coverage.	Data are regularly updated and reflect recent reporting cycles.	Based on official records and standardized data submissions from countries.
			Minority Rights Group International. Retrieved May and June 2018 from:http://minorityrights.org/directory/	3	3	3	2	3	3
				Provides general information on indigenous populations and issues, but not uniformly detailed across countries or sectors.	Based on publicly accessible reports, with unclear methodological consistency or validation protocols.	Lacks detailed occupational or sector-specific risk data.	Covers many countries but with uneven data depth across regions.	Last update from 2018; unclear if systematically maintained.	Based on narrative descriptions with limited verifiability and structured data.
			Minorities Rights Group International. Retrieved May and June 2018 from:http://peoplesunderthreat.org	3	3	3	2	3	3
				Covers many at-risk populations, but may not be exhaustive or frequently updated.	Methodology based on qualitative expert assessments, not fully transparent.	Broad categorization, not specific to activities or sectors.	Wide international scope but more emphasis on known conflict zones.	Last available update in 2018, not recently maintained.	Limited detail on sources and statistical validation.
**3A Indigenous Rights**	Indigenous Sector Issues Identified	The identification of economic sectors with documented conflicts or negative impacts involving Indigenous Peoples, based on external reports.	International Work Group for Indigenous Affairs (2017). *The Indigenous World 2017*. Retrieved April 2018 from: ISBN: 978-87-92786-72-2	3	3	3	2	4	3
				Rich in narrative detail, but coverage and indicators vary between countries.	Compilation from diverse sources with editorial oversight, but lacks methodological uniformity.	Describes indigenous challenges without economic activity breakdown.	Global coverage, but greater emphasis on high-profile regions.	Data collected and reported for 2017, limited updates since.	Descriptive and qualitative, not statistically grounded.
			World Health Organization. (2009). *World Health Statistics 2009*. Retrieved from:http://www.who.int/gho/publications/world_health_statistics/en/index.html	3	3	3	2	5	3
				Provides basic health and demographic stats, but limited focus on indigenous data.	Based on WHO standards, but not tailored for indigenous issues.	Generic national-level statistics.	International scope, but lacking disaggregation by minority groups.	Data over 15 years old, not reflective of current realities.	Standardized format, but outdated and generalized.
			International Labour Organization. (2009). *OVERVIEW REPORT of the Research Project by the International Labour Organization and the African Commission on Human and Peoples' Rights on the constitutional and legislative protection of the rights of*	2	3	3	2	4	3
				In-depth legislative review in 24 African countries. Comprehensive for the scope but limited in	Rigorous legal analysis, but qualitative and limited to African states.	Not focused on sectoral or occupational divisions.	Focused exclusively on Africa; no global perspective.	Published in 2009; context may be outdated.	Strong in legal detail but lacking empirical metrics.

			indigenous peoples in 24 African countries. Retrieved from: http://www.ilo.org/indigenous/Resources/Publications/WCMS_115929/lang--en/index.htm	geographic coverage.					
	Overall risk of indigenous rights being infringedOverall risk of indigenous rights being infringed	The qualitative assessment of risks related to violations of Indigenous Peoples’ rights in a country, based on country-level reports and international reviews.	International Labour Organization (n.d.). NORMLEX Information System on International Labour Standards. Ratifications of C169 - Indigenous and Tribal Peoples Convention, 1989 (No. 169). Retrieved May 2018 from:http://www.ilo.org/dyn/normlex/en/f?p=NORMLEXPUB:11300:0::NO::P11300_INSTRUMENT_ID:312314	2	2	3	2	2	3
				Covers national-level ratification data on ILO Convention 169 but lacks details on enforcement or broader indigenous rights issues.	Methodologically coherent for treaty monitoring but limited for assessing actual implementation.	Not specific to sectors or technologies—provides general ratification status.	Covers a wide range of countries, but coverage is not fully comprehensive.	Data accessed in 2018, with no indication of frequent updates or monitoring.	Based on official ratifications, reliable but does not reflect contextual variability or enforcement in practice.
			International Labour Organization (n.d.). NATLEX Database of national labour, social security and related human rights legislation. Retrieved May 2018 from:http://www.ilo.org/dyn/natlex/natlex4.listResults?p_lang=en&p_count=102935&p_classification=21&p_classcount=527	3	3	3	2	3	3
				Covers relevant legal frameworks on indigenous rights, but data varies in detail and availability across countries.	Legal content is standardized by ILO, but lacks explicit methodological transparency.	Focus on legal instruments may not fully capture implementation or context-specific realities.	Broad international coverage, but country entries differ significantly in depth.	No consistent update cycle; unclear if data reflects current legislation.	Based on official legal texts, but uneven detail and update frequency reduce reliability.
			United Nations Department of Economic and Social Affairs. (2009). State of the World’s Indigenous Peoples. New York, NY. Retrieved from: http://www.un.org/esa/socdev/unpfii/documents/SOWIP_web.pdf	3	3	3	2	4	3
				Covers key global issues affecting Indigenous Peoples’ rights, but is limited to a descriptive overview and lacks comprehensive country-by-country data.	The methodology is based on a UN expert review approach, but not fully documented in a replicable format.	The report discusses general patterns and risks across sectors but lacks detail on specific occupational or sectoral technologies.	The report includes global coverage with focus on several world regions, especially Latin America and Asia.	The report was published in 2009 and is outdated, with no subsequent editions or updates available.	Data are primarily qualitative and compiled from secondary sources, limiting statistical confidence.
			The World Bank. World Bank Open Data. Retrieved May and June 2018 from:https://data.worldbank.org/	3	3	3	3	3	3
				Provides access to a broad range of socioeconomic and development indicators, some of which may indirectly relate to Indigenous Peoples, but lacks disaggregated data specific to them.	The methodology for indicator generation is standardized and robust; however, data specific to Indigenous Peoples is limited or not clearly separated.	The data platform offers general national-level data and does not account for occupational or technological distinctions.	Global scope with broad country coverage, although not all include Indigenous-related variables.	The platform is regularly updated, but updates related specifically to Indigenous data are inconsistent or unclear.	Data are aggregated and statistically sound, though less useful for specific Indigenous-focused analysis.

			World Bank. (2005). Indigenous Peoples, Poverty and Human Development in Latin America: 1994–2004. Retrieved from:http://web.worldbank.org/WBSITE/EXTERNAL/COUNTRIES/LACEXT/0,,contentMDK:20505834~pagePK:146736~piPK:146830~theSitePK:258554,00.html	3	3	2	3	4	3
				Provides targeted analysis of Indigenous Peoples in Latin America with detailed case data, but not structured for comparative statistical reuse.	Analytical and evidence-based report, but not based on standardized or replicable methodology.	Focuses on broad socioeconomic issues without process- or sector-level technological distinctions.	Regional coverage focused on Latin America with contextual depth.	Published in 2005, data are outdated, and no updates are available.	Descriptive findings and case-based evidence, with moderate reliability for cross-country comparisons.
			World Bank. (2010). Indigenous Peoples, Poverty and Development. Hall, G., & Patrinos, H. Retrieved from:http://siteresources.worldbank.org/EXTINDPEOPLE/Resources/407801-1271860301656/full_report.pdf	3	3	2	3	3	3
				Covers socio-economic outcomes and development trajectories for Indigenous Peoples, but lacks a structured indicator framework.	Developed by recognized experts using reliable sources, but not based on a systematic, standardized data method.	Does not provide data segmented by occupational or technological dimensions.	Focused again on Latin America, offering regional depth but lacking global scope.	Published in 2010, with some continued relevance but not updated since.	Narrative and interpretative content with good credibility, but limited statistical rigor.
**3B Gender Equity**	Overall Gender Inequity in Country	The assessment of gender-based disparities in social and economic indicators, reflecting structural inequalities between men and women in a country.	Social Institutions and Gender Index (SIGI). (2012). https://www.genderindex.org/	2	2	2	2	3	2
				Covers institutional and legal gender discrimination across key domains. Structured with specific variables and sub-indices.	Methodologically robust composite index with documented structure and validation.	Reflects sociocultural and institutional aspects, not technical or sectoral processes.	Global dataset covering over 160 countries.	2014 dataset may not reflect current conditions, though still relevant.	Reliable metrics built from standardized questions and country reports.
			World Economic Forum. (2017). The Global Gender Report 2016. http://www.weforum.org/reports/global-gender-gap-report-2011	1	1	2	1	1	1
				Comprehensive index tracking gender disparities across health, education, economy, and politics.	Strong standardized methodology, globally accepted and annually published.	Societal rather than technical focus; not sector-specific.	Broad country coverage with consistent updates.	Very recent data (2017–2018) ensuring high temporal relevance.	High accuracy from standardized cross-country survey and statistical sources.
			United Nations Development Programme. http://hdr.undp.org/en/content/gender-inequality-index-gii and http://hdr.undp.org/en/composite/GII	1	1	2	1	2	1
				Composite index measuring reproductive health, empowerment, and labor participation.	High methodological rigor from the UNDP, regularly reviewed.	Sociopolitical indicators, not directly tied to technology or industry.	Covers almost all UN member states.	Published in 2015, may have slightly outdated data but still actively referenced.	Data derived from national statistics and UN agencies ensure high precision.
				2	2	2	3	3	2

			The CIRI Human Rights Data Project. http://www.humanrightsdata.com/p/data-documentation	Includes women's political and civil rights indicators. Less extensive in economic participation.	Methodology is structured, but coding based on expert judgments introduces subjectivity.	Reflects rights-based rather than sectoral processes.	Good global coverage but less complete for smaller nations.	Based on 2014 data, not the most recent.	Reasonable reliability, though qualitative scoring can vary by coder.
				2	2	2	2	3	2
			ILO – Key Indicators of the Labor Market (KILM) Database, (2015). Table 4a: Female Employment by Sector http://kilm.ilo.org/kilmnet/	Provides sex-disaggregated employment statistics by sector. Useful but limited to formal labor metrics.	Based on official national labor data; harmonized methods across countries.	Focused on employment, not directly on gender norms or institutional barriers.	Broad country coverage, though uneven data availability.	Last update in 2015; relevance may have diminished for recent analysis.	Reliable labor data, though gaps exist in informal sector estimates.
**3C High Conflict Zones**	Overall High Conflict	The overall risk that a country is affected by armed conflict or political violence, based on international monitoring and reporting systems.		2	2	2	1	2	2
			Heidelberg Institute for International Conflict Research. (2017). Conflict Barometer. Retrieved from: https://hiik.de/2018/02/28/conflict-barometer-2017/?lang=en	The dataset provides detailed conflict classification and typologies, but focuses mainly on political/military conflicts.	Conflict typologies and severity levels are clearly defined and systematically applied.	Covers various types of conflict but lacks sectoral nuance.	Global coverage with national-level data.	Updated annually, this edition reflects the situation in 2017.	Based on systematic classification, but qualitative in nature.
				2	2	3	1	1	2
			The UN Refugee Agency, Global Trends Report (2018). Refugees and International Displaced Persons. Retrieved from: http://www.unhcr.org/en-us/figures-at-a-glance.html	Focused on displacement caused by conflict; does not cover conflict typologies.	Data derived from standardized refugee monitoring systems.	Focused on the consequences (displacement), not on the operational or structural aspects of the conflict.	Comprehensive coverage of conflict-affected countries.	Data reflects current situation for 2018.	Based on country reports and refugee registration data.
				2	2	2	1	2	2
			Center for Systemic Peace. (2017). Global Report on Conflict, Governance and State Fragility. Retrieved from: http://www.systemicpeace.org/globalreport.html	Provides detailed data on conflict episodes, political instability, and governance indicators.	Methodology is documented and consistent but includes composite indicators with normative assumptions.	Reflects systemic characteristics of conflict and governance, not operational conflict typologies.	Global coverage with data for nearly all countries.	Data is updated periodically, 2017 edition may not reflect immediate changes.	Based on validated coding of events and institutional analysis.
				2	2	3	2	2	3
			Minority Rights Group International. (2018). Peoples Under Threat 2018. Retrieved from: https://minorityrights.org/publications/peoplesunderthreat2018/	Covers structural and emerging risks of mass violence based on multiple conflict-related indicators, but limited in real-time conflict tracking.	Methodology is based on a composite risk index combining quantitative and qualitative data; transparent but	Focused on population vulnerability and minority status, not direct military or political conflict events.	Good regional coverage, but some countries have limited or outdated data.	Based on annual reports; reflects the year of publication but may not capture recent escalations.	Uses mixed data sources and expert judgment; may have inconsistencies across countries.

					includes subjective weighting.				
**3D Non-Communicable Diseases**	Overall Non-communicable Diseases and other health risks	The assessment of the population-level burden from non-communicable diseases such as cardiovascular diseases, diabetes, and cancer, reflecting overall health risks.	Global Health Estimates 2016: Deaths by Cause, Age, Sex, by Country and by Region, 2000–2016. Geneva, World Health Organization; 2018. WHO Global Health Observatory (GHO) data. Retrieved from: http://www.who.int/gho/en/	1	1	1	1	2	1
				Provides extensive mortality data for NCDs by cause, sex, and country; covers major relevant variables.	Based on standardized and internationally accepted epidemiological methods.	Reflects a broad spectrum of health conditions related to technology and healthcare systems.	Global in scope, with country-level detail and harmonized data formats.	Data reflects the 2016 reference year; slight time lag in availability.	Based on official statistics and health surveillance systems with rigorous quality control.
			Prevalence of undernourishment (% of population). Retrieved from: https://data.worldbank.org/indicator/SN.ITK.DEFC.ZS.	3	2	2	2	2	2
				Focused solely on undernourishment; relevant but does not fully represent NCD burden.	Uses established FAO methodologies in collaboration with the World Bank, ensuring consistency.	Related to food access and nutrition but does not directly reflect medical or technological health conditions.	Covers a large number of countries, but data quality may vary depending on national reporting.	Regularly updated, though some countries may have time lags in data.	Based on national survey and food balance estimates, with moderate uncertainty in low-data regions.
**3E Communicable Diseases**	Age-standardized MRs from communicable diseases (per 100,000 population)	The age-standardized mortality rate attributed to communicable diseases per 100,000 people, indicating the impact of infectious disease burden.	WHO Global Health Observatory (GHO) data. Retrieved from http://www.who.int/gho/en/	1	1	1	1	1	1
				Covers mortality from a broad range of communicable diseases with age-standardization, allowing international comparability.	Uses standard WHO methodology based on ICD coding and statistical modelling for consistency across countries.	Reflects current medical classifications and health reporting protocols.	Global coverage with consistent country-level data.	Frequently updated with data series reflecting recent years.	Derived from official national health statistics and epidemiological models with high reliability.
	Cases of HIV (per 1000 adults 15-49 years)	The estimated number of adults living with HIV per 1000 population aged 15 to 49, reflecting national prevalence levels.	WHO Global Health Observatory (GHO) data. Retrieved from http://www.who.int/gho/en/	1	1	1	1	1	1
				Provides comprehensive HIV prevalence estimates for adults aged 15–49 using consistent indicators across countries.	Based on standardized WHO/UNAIDS modelling and surveillance systems, harmonized globally.	Aligned with up-to-date public health technologies and diagnostic protocols.	Covers nearly all countries with harmonized national data.	Regularly updated with the most recent annual estimates.	High-quality estimates derived from surveillance, clinical reports, and validated models.
	Cases of Tuberculosis (per 100,000 population)	The incidence of tuberculosis per 100,000 inhabitants, used to assess the population's exposure to a major communicable disease.	WHO Global Health Observatory (GHO) data. Retrieved from http://www.who.int/gho/en/	1	1	1	1	1	1
				Provides global estimates of TB incidence per 100,000 population with standardized indicators and complete country-level data.	Based on harmonized surveillance systems and WHO guidelines for TB case detection and reporting.	Reflects current diagnostic and treatment protocols widely used in TB control.	Comprehensive geographic coverage of WHO member states.	Regularly updated with yearly estimates reflecting current epidemiological data.	Uses high-quality national surveillance, laboratory-confirmed cases, and modeling approaches.
				2	2	2	1	1	2

	Dengue Fever, Incidence rate (per 100,000 population)	The number of reported dengue fever cases per 100,000 people, used to evaluate the prevalence and risk of vector-borne illness.	WHO Global Health Observatory (GHO) data. Retrieved from http://www.who.int/gho/en/	Data available for most countries, though quality and completeness may vary by national reporting capacity.	Uses WHO case definitions and national surveillance systems, but differences in diagnostic and reporting practices exist.	Reflects general detection and reporting methods for vector-borne diseases, but some countries lack advanced diagnostics.	Broad coverage of dengue-endemic and non-endemic countries.	Data updated regularly; incidence rates reflect current reporting cycles.	Based on reported cases, which may be undercounted due to asymptomatic infections or underreporting in some regions.
				2	2	2	1	1	2
	Notified cases of Malaria (per 100,000 population)	The number of malaria cases reported by health authorities per 100,000 individuals, used to monitor exposure and disease control efforts.	WHO Global Health Observatory (GHO) data. Retrieved from http://www.who.int/gho/en/	Data on malaria incidence are widely reported, especially in endemic regions, but quality may vary depending on surveillance infrastructure.	WHO standardizes case definitions and data collection, though implementation may differ across countries.	Represents the typical surveillance and diagnostic capabilities, which may be limited in rural or remote areas.	Includes nearly all malaria-endemic countries.	Data is updated annually and reflects current trends.	Relies on national reporting; underreporting may occur in regions with limited access to diagnostics or healthcare.
				2	2	2	1	2	2
**4A Legal System**	Overall Fragility in Legal System	The qualitative assessment of the strength and functionality of a country's legal system, including the rule of law, judicial independence, and access to justice.	World Bank. (2011). *Worldwide Governance Indicators*. http://info.worldbank.org/governance/wgi/sc_country.asp	Covers legal system aspects (rule of law, judicial independence) but not all dimensions of fragility.	Aggregated from multiple data sources using a consistent methodology, though it includes perception-based data.	Reflects general institutional conditions, not specific legal mechanisms or infrastructure.	Covers over 200 countries and territories.	Based on 2011 data; may not reflect current governance conditions.	Combines perception surveys and expert assessments; methodological transparency, but limited primary data.
				2	2	3	2	3	3
			Bertelsmann Stiftung. (2012). *Transformation Index BTI*. http://www.bertelsmann-stiftung.de	Covers core elements of rule of law and legal functionality, but with emphasis on political transformation.	Based on expert assessments following structured methodology, with cross-country comparability.	The assessment covers broad governance structures and transformation indicators, but lacks detail on legal technologies or institutional mechanisms.	Includes a wide set of countries, but less comprehensive than UN or WB datasets.	The data refers to the 2012 edition, which may not accurately reflect the current state of legal systems in many countries.	Expert-based evaluation with clear guidelines, but remains qualitative and potentially subjective in interpretation.
				2	2	3	2	3	3
			The CIRI Human Rights Data Project. (2010). http://ciri.binghamton.edu	Covers key legal and civil rights dimensions relevant to rule of law, though not all aspects of legal system functionality.	Based on standardized coding by multiple coders with inter-coder reliability checks.	Focuses on civil and legal rights, but does not address institutional or technological structures of legal systems.	Includes over 190 countries, though depth of coverage may vary.	Last available update from 2010, limiting its reflection of recent legal developments.	Uses qualitative coding of expert-reviewed reports; moderate transparency and subjectivity.
				2	2	3	2	3	3

			Global Integrity Report. (2011). http://www.globalintegrity.org/report	Covers a broad set of governance-related legal indicators but may not fully encompass the operational aspects of legal systems.	Employs peer-reviewed scoring mechanisms and standardized frameworks, but with variations in source interpretation.	Focuses on outcomes and perceptions rather than legal system infrastructure or technology.	Covers a significant number of countries, but some regions are underrepresented or inconsistently updated.	The 2011 edition reflects the context of over a decade ago and does not include ongoing updates.	Based on expert assessments with some transparency, though scores may be influenced by subjective judgment.
			The World Justice Project. (2011). http://www.worldjusticeproject.org	2	2	2	1	3	2
				Covers multiple dimensions of the legal system, including constraints on government powers, absence of corruption, and access to justice, but may omit detailed data on judiciary effectiveness.	Uses structured and transparent methodology combining expert and population surveys, though subjectivity and comparability issues remain.	Captures legal system performance rather than infrastructure or specific technologies.	Global coverage with data from over 100 countries.	Data is from 2011; outdated with no clear annual continuity.	Combines perception-based and factual survey data with transparent sources, but subject to some interpretation bias.
**4B Corruption**	Overall Corruption	The assessment of the prevalence and perception of corruption within a country, based on international indices and reports.	World Bank. (2016). Worldwide Governance Indicators. Retrieved from: http://info.worldbank.org/governance/wgi/#home	1	1	2	1	1	2
				Provides broad coverage of corruption-related governance aspects through multiple indicators and composite indexes.	Uses a rigorous and transparent methodology, aggregating data from over 30 sources including expert assessments and surveys.	Focuses on institutional performance and public sector practices, not directly tied to specific technologies.	Covers over 200 countries and territories, ensuring extensive comparability.	Data is available annually and is up-to-date for 2016, with regular releases.	Based on multiple data sources with clear documentation, though perception-based components introduce some uncertainty.
			World Economic Forum. (2017–2018). The Global Competitiveness Report 2017–2018. Geneva, Switzerland. Retrieved from: http://reports.weforum.org/global-competitiveness-index-2017-2018/competitiveness-rankings/#series=BRIBEID	2	2	2	2	1	2
				Focuses on specific aspects of corruption perception (e.g., bribe payments), but not comprehensive across all sectors or forms.	Combines executive opinion surveys and hard data using a transparent framework, though dependent on subjective perceptions.	Captures corruption perception in economic environments, not necessarily across all institutional or technological domains.	Includes a wide range of countries (over 130), but some variation in data availability and quality.	Reflects the status for the year 2017–2018, with annual updates.	Uses perception-based data from national business leaders, introducing variability and potential bias.
			Transparency International. (2017). Retrieved from: https://www.transparency.org/news/feature/corruption_perceptions_index_2017	2	2	2	1	1	2
				Covers corruption perception across public sectors, but does not address private or informal contexts in depth.	Methodology integrates multiple data sources (expert assessments, business surveys)	Focused on institutional corruption; does not fully reflect technological or	Broad global coverage (180 countries), allowing consistent cross-country comparison.	Reflects the situation in 2017 with annual reporting, supporting temporal relevance.	Based on a composite of subjective data, which introduces some uncertainty in accuracy.

					in a consistent and documented way.	sector-specific corruption risks.			
**5A Access to Drinking Water**	% Total Access to an Improved Source of Drinking Water	The percentage of the population with access to improved drinking water sources, such as piped water or protected wells.	World Health Organization and UNICEF. (2017). Progress on drinking water, sanitation and hygiene: 2017 update and SDG baselines. Retrieved from: http://washdata.org/report/jmp-2017-report-final	1	1	2	1	1	2
				Comprehensive data on access to improved drinking water, disaggregated by region and urban/rural setting.	Standardized methodology developed jointly by WHO and UNICEF ensures high consistency and comparability.	Reflects access types (piped, protected wells, etc.) but not quality or performance of technologies used.	Global coverage with data from over 190 countries.	Data reflects the 2017 baseline for SDG monitoring, ensuring temporal relevance.	Data is based on national household surveys and administrative records, which may vary in quality and frequency.
**5B Access to Sanitation**	% Total Access to an Improved source of Sanitation	The percentage of the population using improved sanitation facilities, including toilets connected to sewer systems or septic tanks.	World Health Organization and UNICEF. (2017). Progress on drinking water, sanitation and hygiene: 2017 update and SDG baselines. Retrieved from: http://washdata.org/report/jmp-2017-report-final	1	1	2	1	1	2
				Extensive and structured data on population access to improved sanitation, with regional and rural/urban disaggregation.	WHO/UNICEF Joint Monitoring Programme provides consistent definitions and standardized methodology across countries.	Captures presence of improved sanitation infrastructure, but not necessarily its functionality or maintenance.	Broad global coverage including data from most countries.	Reflects current conditions as of the 2017 baseline, aligned with SDG monitoring.	Based on national-level survey and census data, with some variability in frequency and reporting standards.
**5C Children out of School**	Percent of Children Out of Primary School, total	The proportion of primary-school-aged children not enrolled in school, used to assess educational access and exclusion risks.	UNESCO. Institute for statistics(2017). https://tellmaps.com/uis/oosc/#!/tellmap/-528275754	1	1	2	1	1	2
				Provides disaggregated statistics on out-of-school children by country, age, and gender, using standardized international definitions.	UNESCO Institute for Statistics follows rigorous and consistent education monitoring methodology.	Tracks school enrollment status, but not detailed data on attendance or informal education.	Global coverage across low-, middle-, and high-income countries.	Based on the most recent data available as of 2017, aligned with SDG 4 indicators.	Derived from national administrative data and household surveys, with some variability in data quality and frequency.
**5D Access to Hospital Beds**	Number of Hospital Beds per 1000 population	The number of available hospital beds per 1,000 people, used as a proxy for access to healthcare infrastructure.	2018 World Development Indicators Report, The World Bank https://data.worldbank.org/indicator/SH.MED.BEDS.ZS	2	2	2	1	1	2
				Provides national-level data on hospital bed availability, but may not capture differences in public vs private sector or rural vs urban contexts.	Data compiled using harmonized definitions across countries; methodology is appropriate but dependent on national reporting standards.	Measures infrastructure availability (beds) but does not reflect actual access, usage, or service quality.	Broad global coverage with data from both developed and developing countries.	Data reflects most recent reporting from countries around 2017–2018.	Based on national health administrative sources, though reporting intervals and definitions can vary slightly.
			Central Intelligence Agency (CIA). Library The World Fact Book https://www.cia.gov/library/publications/resources/the-world-factbook/fields/360.html	3	3	2	2	3	3
				Coverage varies by country and some entries are outdated or estimated; data not always sourced from health authorities.	Methodologies are not always disclosed; aggregation of diverse sources can lead to inconsistency.	Captures number of beds as an infrastructure proxy, but not linked to healthcare delivery quality or access.	Good global reach but variable granularity across countries.	Some data may be outdated or not regularly updated; no consistent time series.	Compiled from secondary sources; limited detail on source accuracy and definitions.

				2	2	2	3	2	2
			OECD Data Hospital Beds https://data.oecd.org/healtheqt/hospital-beds.htm	Covers hospital beds consistently across OECD countries with structured definitions.	Uses standardized OECD definitions and data collection processes, although methods can differ slightly between countries.	Measures physical hospital capacity, not functional service quality or access.	Limited to OECD members, excluding many low-income countries.	Updated periodically; some countries may lag in reporting by 1–2 years.	Data sourced from national health systems and ministries; generally reliable and consistent.
**5E Smallholder v Commercial Farms**				2	2	2	2	3	2
	Largeholdings Land % < x hectares	The percentage of land held by large commercial farms, defined by exceeding a threshold area of cultivated land.	FAO. (2010). World Census of Agriculture. Retrieved from: http://www.fao.org/docrep/013/i1595e/i1595e00.htm	Covers landholding size across many countries, though not always for the same year or survey cycle.	Based on national agricultural censuses harmonized by FAO; consistent but not always fully standardized.	Reflects large-scale farms in terms of land area, though not detailed by mechanization or production system.	Wide country coverage through national reports.	Data years vary greatly by country (ranging from 1990s to 2007).	Data drawn from national statistical reports and census methods.
				2	2	3	2	1	2
	Overall risk of Freedom of Association	The risk that workers on smallholder or commercial farms face limitations on their rights to form or join organizations, bargain collectively, or strike, particularly in agricultural contexts marked by informal labor or insecure land tenure.	ITUC. (2018). ITUC Global Rights Index – The World's Worst Countries for Workers. https://www.ituc-csi.org/ituc-global-rights-index-2018	Covers key dimensions of labor rights, including freedom of association, but not always disaggregated by sector or type of worker (e.g., smallholder vs commercial farm).	Based on a consistent rating system across countries using qualitative and legal sources.	Does not differentiate technological contexts such as industrial vs traditional farming.	Covers a wide range of countries with comparable scoring.	Most recent version (2018) reflects contemporary labor situations.	Based on verified country-level data from multiple labor and human rights sources.
				2	2	3	2	4	2
	Percentage of commercially-owned farms in country	The proportion of farms that are owned and operated by commercial entities, reflecting the industrialization of agriculture.	ILO Laborsta. (2011). Economically Active Population by Industry and Status in Employment – Table 1C. http://laborsta.ilo.org/data_topic_E.html	Provides employment data by status and sector, including categories related to commercial farming, though not always explicitly distinguished.	Based on ILO's statistical standards with consistent categorization across countries.	Lacks specificity on farming systems or production scale, which limits representativeness for commercial farms.	Global scope but limited coverage for some countries or outdated entries.	Data refers to 2011 and is not regularly updated.	Based on official labor force statistics and government records.
				2	2	3	2	4	2
	Percentage of family-owned farms in country	The proportion of farms that are owned and operated by family units, indicating the prevalence of smallholder agricultural systems.	ILO Laborsta. (2011). Economically Active Population by Industry and Status in Employment – Table 1C. http://laborsta.ilo.org/data_topic_E.html	Covers employment by industry and employment status, which may include family labor, but not always directly indicates family farm ownership.	Uses standardized methodology aligned with ILO labor classifications.	Does not distinguish farm ownership structures clearly, reducing representativeness for family-based agriculture.	Global coverage, but with gaps or outdated entries for some countries.	Data refers to 2011 and lacks updates in recent years.	Derived from national labor statistics with recognized methodological basis.

	Smallholdings Land % < x hectares	The percentage of land held by small farms operating under a defined size threshold, often linked to subsistence or semi-subsistence farming.	Nagayets, O. (2005). Small Farms: Current Status and Key Trends. http://www.ifpri.org/publication/future-small-farms	4	4	4	4	5	3
				The report presents general trends and characteristics of small farms, but lacks disaggregated or structured data per country.	Methodology is based on literature review and secondary sources with no standardized approach across datasets.	Technological detail is not provided; general references to small-scale farming without production system specification.	Geographic coverage is broad but uneven and not consistent by country.	Data is from or before 2005 and has not been updated.	Data is synthesized from multiple references with moderate reliability.
			Eurostat. (2011). http://epp.eurostat.ec.europa.eu/portal/page/portal/agriculture/data/database	2	2	3	2	3	2
				Provides structured and comprehensive data for EU countries, though limited to the European region.	Methodology is based on standardized agricultural surveys and classification criteria defined by Eurostat.	Refers to land size and ownership, without specifying production systems or technologies.	Covers all EU member states, but not globally representative.	Data from 2011 is somewhat outdated, though historical series are accessible.	Based on harmonized surveys with high reliability for the covered countries.

Appendix S.4 – Uncertainty score and uncertainty level of social indicators

Table S10: Uncertainty score and uncertainty level of social indicators.

Impact Subcategory	Indicator	Uncertainty Score	Uncertainty Value	Uncertainty Level
**Wage Assessment**	Risk that Avg Wage is Below Country Minimum Wage	2.17	2	Medium
	Risk that Sector Avg Wage is below Living Wage	2.50	2	Medium
	Risk that Sector Avg Wage is below Sweatered Wage	3.00	2	Medium
**Poverty**	Percent of population living under the relevant poverty line	1.17	1	Low
**Child Labor**	Risk of child labor by sector (qualitative)	3.08	3	High
**Forced Labor**	Overall Forced Labor in Country	2.17	2	Medium
**Excessive Working Time**	Percent of Population working >X hrs. per week, >60 hrs. per week	1.33	1	Low
**Freedom of Association**	Overall risk of Freedom of Association	2.58	2	Medium
**Migrant Labor**	Evidence of Risk to Migrant Workers - Qualitative	2.83	2	Medium
**Social Benefits**	Overall risk of inadequate social benefits	2.67	2	Medium
**Labor Laws Conventions**	Number of Labor Laws by Sector	2.67	2	Medium
**Discrimination**	Prevalence of discrimination in the workplace (qualitative)	3.17	3	High
**Unemployment**	Unemployment percentage at sector level	2.17	2	Medium
**Occupational Toxics and Hazards**	Disability-adjusted life years due to occupational-related Lung Cancer	3.17	3	High
	Overall Occupational Cancer Risk - loss of life (DALYs)	3.17	3	High
	Overall Occupational Noise Exposure Risk	3.17	3	High
**Injuries and Fatalities**	Fatal injuries by sector	1.83	2	Medium
	Non-Fatal Work Related injuries by sector	1.83	2	Medium
**Indigenous Rights**	Indigenous Sector Issues Identified	2.93	2	Medium
	Overall risk of indigenous rights being infringed	2.83	2	Medium
**Gender Equity**	Overall Gender Inequity in Country	1.83	2	Medium
**High Conflict Zones**	Overall High Conflict	1.96	2	Medium
**Non-Communicable Diseases**	Overall Non-communicable Diseases and other health risks	1.67	2	Medium

Impact Subcategory	Indicator	Uncertainty Score	Uncertainty Value	Uncertainty Level
**Communicable Diseases**	Age-standardized MRs from communicable diseases (per 100,000 population)	1.00	1	Low
	Cases of HIV (per 1000 adults 15-49 years)	1.00	1	Low
	Cases of Tuberculosis (per 100,000 population)	1.00	1	Low
	Dengue Fever, Incidence rate (per 100,000 population)	1.67	2	Medium
	Notified cases of Malaria (per 100,000 population)	1.67	2	Medium
**Legal System**	Overall Fragility in Legal System	2.27	2	Medium
**Corruption**	Overall Corruption	1.61	2	Medium
**Access to Drinking Water**	% Total Access to an Improved Source of Drinking Water	1.33	1	Low
**Access to Sanitation**	% Total Access to an Improved source of Sanitation	1.33	1	Low
**Children Out of School**	Percent of Children Out of Primary School, total	1.33	1	Low
**Access to Hospital Beds**	Number of Hospital Beds per 1000 population	2.17	2	Medium
**Smallholder vs. Commercial Farms**	Largeholdings Land % < x hectares	2.17	2	Medium
	Overall risk of Freedom of Association	2.00	2	Medium
	Percentage of commercially-owned farms in country	2.50	2	Medium
	Percentage of family-owned farms in country	2.50	2	Medium
	Smallholdings Land % < x hectares	3.17	3	High

Appendix S.5 – Descriptive statistics for social impact subcategories under stochastic simulation

Table S11: Social impact descriptive statistics for the car dashboard.

		Q1 (25%)	Median	Q3 (75%)
Car dashboard	Single Score			
	Cellulose	1057.5	1167.63	1280.36
	ABS	623.01	691.51	762.79
	Corruption			
	Cellulose	11.92	17.81	22.91
	ABS	6.37	10.01	13.11
	Occ Tox & Haz			
	Cellulose	12.56	16.67	21.47
	ABS	7.11	9.35	11.87
	Injuries & Fatalities			
	Cellulose	9.06	12.43	15.99
	ABS	5.37	7.17	8.91
	Legal System			
	Cellulose	9.14	12.6	16.1
	ABS	6.05	8.48	10.79
	High Conflict Zones			
	Cellulose	12.56	19.12	24.87
	ABS	7.58	11.21	14.73

Table S12: Social impact descriptive statistics for the ship counter bar.

		Q1 (25%)	Median	Q3 (75%)
Ship counter bar	Single Score			
	Cellulose	1114.31	1232.03	1353.28
	Gypsum	13424.18	14806.28	16221.01
	Corruption			
	Cellulose	12.44	19.16	24.2
	Gypsum	155.3	225.4	282.97
	Occ Tox & Haz			
	Cellulose	13.4	17.8	23
	Gypsum	157.95	209.05	267.08
	Legal System			
	Cellulose	9.89	13.25	17.05
	Gypsum	113.38	161.1	209.46
	Injuries & Fatalities			
	Cellulose	10.05	13.37	16.93
	Gypsum	140.03	153.24	181.87
	High Conflict Zones			
	Cellulose	12.7	20.35	26.52
	Gypsum	219.35	241.8	272.59
